# Quantifying the potential value of entomological data collection for programmatic decision-making on malaria control in sub-Saharan African settings

**DOI:** 10.1186/s12936-025-05251-7

**Published:** 2025-01-30

**Authors:** Nora Schmit, Hillary M. Topazian, Matteo Pianella, Giovanni D. Charles, Peter Winskill, Penelope A. Hancock, Ellie Sherrard-Smith, Katharina Hauck, Thomas S. Churcher, Azra C. Ghani

**Affiliations:** 1 MRC Centre for Global Infectious Disease Analysis, Imperial College London, London, UK; 2 Department of Economics, Stockholm University, Stockholm, Sweden; 3 MRC Centre for Global Infectious Disease Analysis, Jameel Institute, Imperial College London, London, UK; 4 Liverpool School of Tropical Medicine, Liverpool, UK

**Keywords:** Entomological surveillance, Malaria, Mathematical modelling, Health economics

## Abstract

**Background:**

The availability of many tools for malaria control leads to complex decisions regarding the most cost-effective intervention package based on local epidemiology. Mosquito characteristics influence the impact of vector control, but entomological surveillance is often limited due to a lack of resources in national malaria programmes.

**Methods:**

This study quantified the monetary value of information provided by entomological data collection for programmatic decision-making using a mathematical model of *Plasmodium falciparum* transmission. The 3-year impact and cost of various intervention packages was simulated in different sub-Saharan African settings, including combinations of scaling-up insecticide-treated nets (ITN), switching to next-generation ITNs, and a treatment and prevention package. The DALYs averted and their net monetary benefit were compared at different cost-effectiveness thresholds and the value of resolving uncertainty in entomological model parameters was calculated.

**Results:**

Across transmission settings and at cost-effectiveness thresholds over US$170 per DALY averted, the most cost-effective intervention package was switching to and scaling up pyrethroid-pyrrole ITNs combined with the treatment and prevention package. The median expected value of perfect information on the entomological indicators was US$0.05 (range 0.02–0.23) and US$0.17 (range 0.09–1.43) per person at risk at thresholds of US$75 and US$1000 per DALY averted, respectively. This represented less than 2% of the net monetary benefit of implementing the most cost-effective intervention package. Value of information estimates at cost-effectiveness thresholds over US$250 were higher than current investments into entomological monitoring by the US President’s Malaria Initiative.

**Conclusions:**

These results suggest that entomological data collection should not delay implementation of interventions with demonstrated efficacy in most settings, but that sustained investments into and use of entomological surveillance are nevertheless worthwhile and have broad value to national malaria programmes.

**Supplementary Information:**

The online version contains supplementary material available at 10.1186/s12936-025-05251-7.

## Background

Large-scale deployment of insecticide-treated mosquito nets and other control measures over the last two decades has substantially reduced malaria cases. However, progress has stalled since 2017 and malaria still constitutes an important cause of mortality, especially in sub-Saharan Africa [1]. Possible contributing factors to this include the widespread resistance to pyrethroid insecticides in mosquitoes [2], residual transmission due to vector behaviours not targeted by standard vector control products such as biting outside of the time when people are indoors and in bed [3], and gaps in effective coverage and durability of insecticide-treated nets (ITNs) [4]. Novel tools have been developed to counter these emerging threats. In areas with pyrethroid-resistant mosquitoes, the World Health Organization (WHO) has recommended the use of new classes of ITNs, first pyrethroid-PBO nets treated with a pyrethroid and the synergist piperonyl butoxide and, since 2023, the dual-active-ingredient pyrethroid-pyrrole nets [5]. Next-generation long-lasting insecticides have also become available for indoor residual spraying (IRS) [6], and the first vaccine against malaria, RTS,S/AS01, is being rolled out in moderate and high transmission regions of sub-Saharan Africa [1].

With the availability of more tools, resource allocation decisions and selection of the most appropriate intervention package in a given setting have become more complex [7, 8]. The WHO recommends that intervention use should be optimized based on effectiveness and value for money, and tailored to local settings [9]. A good understanding of all aspects of the local epidemiological context is therefore essential and surveillance systems, including entomological surveillance, are considered a core intervention in malaria control and elimination programmes [9]. Entomological surveillance involves regular collection of data on mosquito characteristics and the effectiveness of vector control: key entomological indicators include vector species identification, their relative abundance, anthropophilic blood feeding habits, vector biting behaviour (e.g. the propensity to blood-feed at a time when people are outdoors), the level of insecticide resistance, malaria infection rates, larval habitats identification, and the coverage, durability and efficacy of vector control interventions [10]. Data on these indicators can then be used to make decisions, for example for risk stratification and on subnational allocation of different classes of ITNs, or selecting districts for IRS implementation that have greater densities of mosquito species which rest indoors [11, 12]. Vector bionomics are highly diverse, sometimes varying substantially across small geographical scales, and changing over time. Nevertheless, published entomological data on mosquito and human behaviour are sparse [8] and entomological surveillance is often not prioritised due to a lack of resources in national malaria programmes [13].

Two randomized controlled trials have demonstrated the efficacy and cost-effectiveness of dual-active-ingredient relative to pyrethroid-only ITNs in areas with pyrethroid-resistant mosquitoes [14, 15], but the level of protection can vary due to local factors and it is not feasible to conduct randomized controlled trials across all settings [16]. Uncertainty persists about the relationship between phenotypic resistance levels and the epidemiological impact of ITNs [12, 17]; use of pyrethroid-only ITNs delivers some protection against malaria even in the presence of resistance [18], which could complicate decisions to adopt more expensive new nets. Additionally, while ITNs have traditionally represented the most cost-effective intervention, insecticide resistance could also affect the relative cost-effectiveness of interventions other than vector control [19]. Previous cost-effectiveness analyses have often considered individual interventions as opposed to intervention packages [6, 14, 20, 21]. The uncertainty underlying decisions between various combinations of interventions, including scale-up of existing interventions, and the local epidemiology has, therefore, not been fully characterized [12].

In using entomological data to inform the optimal selection of interventions, its value for money and potential delays in deployment of effective tools whilst entomological data is collected need to be considered. Value of information (VOI) analysis is a technique to estimate the value of collecting additional information to reduce the uncertainty surrounding decision-making [22–24]. In this paper, this method is applied together with a published mathematical model of malaria transmission to determine the most cost-effective intervention package and quantify the potential monetary value of entomological data collection for programmatic decision-making in different African settings. The aim was to determine to which settings and which entomological indicators surveillance should be targeted and to compare the estimated value of information to current investments into entomological monitoring.

## Methods

### Mathematical model and interventions

A previously developed individual-based mathematical model of *Plasmodium falciparum* malaria transmission was used to simulate the impact of various interventions in a wide range of transmission settings across sub-Saharan Africa (Appendix pp.3–18) [25, 26]. The model was parameterized using parasite prevalence and clinical and severe disease incidence data from different sub-Saharan African settings, by accounting for the acquisition of immunity, seasonality in transmission, mosquito population dynamics and biting behaviour. The model represents the infection process in humans, including the development of asymptomatic infection and clinical disease, and treatment with first-line artemisinin-based combination therapy (ACT). It also incorporates the mosquito life cycle through a compartmental model from egg to adult. Adult female mosquitoes can acquire malaria infection when biting an infectious human. In addition to treatment, population-level interventions recommended by the WHO were modelled, including the different classes of ITNs, IRS, seasonal malaria chemoprevention (SMC) and age-based vaccination with the RTS,S vaccine.

### Transmission settings and model scenarios

36 baseline settings were modelled to represent the variety of transmission settings across Africa, focusing on those with population-wide pyrethroid-only ITN usage (Appendix p.19). Baseline settings comprised four transmission intensities (mean *P. falciparum* parasite prevalence in 2–10 year olds (*Pf*PR_2-10_) over a 3-year period of 5%, 10%, 20% and 40%), three seasonality profiles (perennial based on Central Africa, seasonal based on West Africa coastal regions and highly seasonal based on the Sahel region) and three baseline pyrethroid-only ITN usage levels (20%, 40% and 60%). All settings were assumed to have 45% treatment coverage, and SMC at 85% coverage was in place in highly seasonal settings at baseline per WHO recommendations [5, 27].

In each setting, the effect of 17 different intervention packages were simulated over a 3-year time horizon to align with funding periods [28] and to capture a full ITN distribution cycle, during which ITN use and efficacy wanes over time after mass distribution (Table 1). Intervention options were based on WHO recommendations [5], and consisted of all possible combinations of switching from pyrethroid-only to pyrethroid-PBO or pyrethroid-pyrrole nets at the baseline usage level, increasing ITN usage by 20%, implementing a setting-specific treatment and prevention package, and introducing IRS at 80% coverage. The treatment and prevention package includes an increase in diagnosis and treatment coverage to 65%, introduction of SMC at 85% coverage (in seasonal settings) and introduction of age-based RTS,S vaccination at 85% coverage (in settings with *Pf*PR_2–10_ ≥ 10%). Details are provided in the Appendix p.19 and previous publications [19].

**Table 1:** Overview of intervention scenarios

	Component interventions					
		ITN switch to pyrethroid-PBO	ITN switch to pyrethroid-pyrrole	ITN scale-up	Treatment and prevention package	IRS
Intervention package	1	X				
	2		X			
	3			X		
	4				X	
	5	X		X		
	6		X	X		
	7	X			X	
	8		X		X	
	9			X	X	
	10	X		X	X	
	11		X	X	X	
	12					X
	13	X				X
	14		X			X
	15				X	X
	16	X			X	X
	17		X		X	X

The intervention packages comprise combinations of switching the insecticide in insecticide-treated nets (ITNs) from pyrethroid-only to pyrethroid-PBO or pyrethroid-pyrrole, scaling up ITN usage by 20 percentage points, and implementing a setting-specific treatment and prevention package (case A: package 1–11). Case B (packages 1–17) additionally includes introduction of indoor residual spraying (IRS) at 80% coverage. The treatment and prevention package is tailored to the specific setting and includes increased access to diagnosis and treatment (from 45% to 60% coverage), seasonal malaria chemoprevention (SMC) (85% coverage, in seasonal settings) and RTS,S vaccination (85% coverage, in settings of *Pf*PR_2–10_ ≥ 10%). All intervention scenarios were compared to the corresponding baseline setting with existing ITN use, 45% treatment coverage, and, for highly seasonal settings, SMC at 85% coverage

Introduction of IRS was considered as an alternative to ITN scale-up [5]. IRS is usually targeted to more localized areas because of logistical and affordability challenges [7]. Due to its much higher cost (Fig. S2.1), the analysis was divided into two decision cases: case A for a decision between all interventions packages except those including IRS (1–11), and case B for all interventions including IRS (1–17).

### Sources of entomological parameter uncertainty

**Fig. 1:**
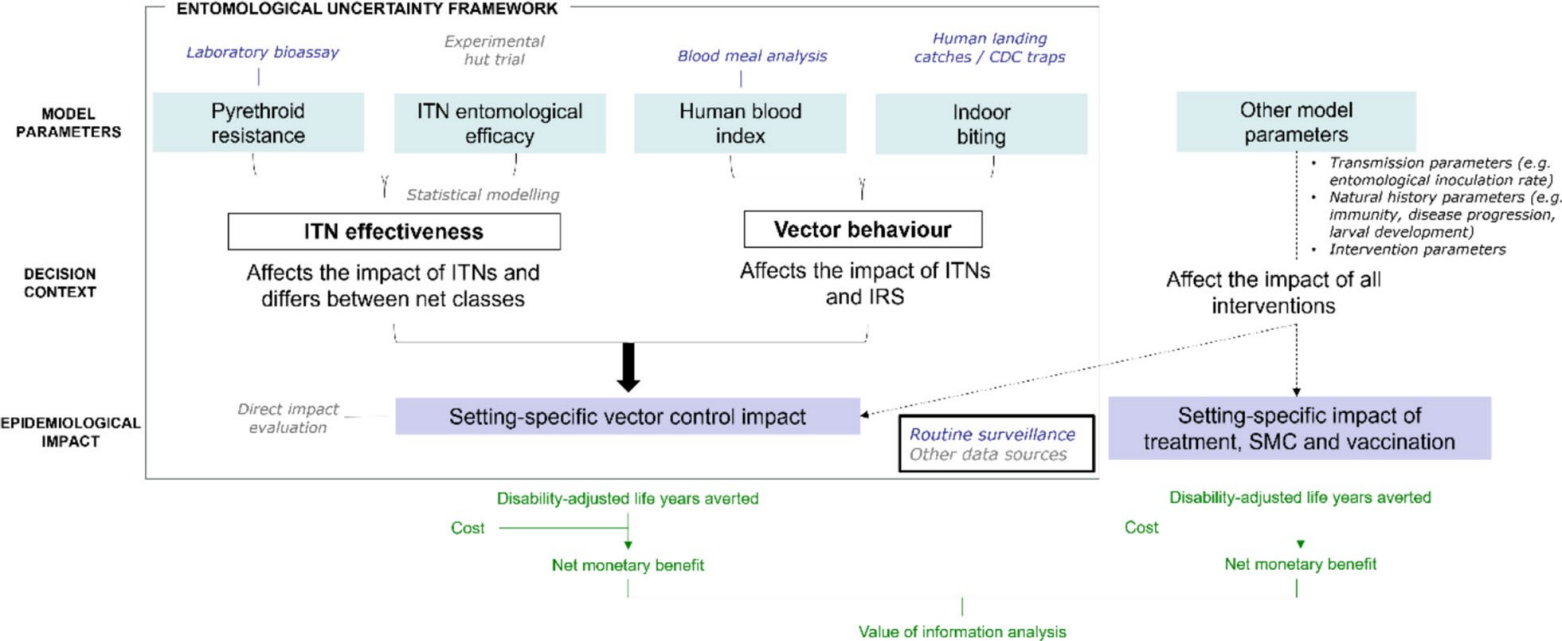
Schematic of entomological indicators in the model and potential data sources. Uncertainty in the four entomological indicators affects the impact of different classes of ITN and IRS relative to all other interventions in the model. Data on these indicators can be obtained through routine entomological surveillance and other sources like experimental hut trials and statistical modelling, while the setting-specific impact of vector control can also be assessed directly using (post-implementation) impact evaluations. Estimates of indoor biting behaviour also require data on human activity in addition to entomological field studies

VOI analysis estimates the value of eliminating uncertainty in model parameters through further data collection. Uncertainty in four key entomological parameters influencing the impact of interventions in model simulations was accounted for: pyrethroid insecticide resistance, the entomological efficacy of ITNs in relation to resistance levels, and the propensity for anthropophagic and endophilic biting behaviour in the local mosquito population (Table 2, Fig. 1).

**Table 2:** Sources of entomological parameter uncertainty in the analysis

	Entomological indicator	Model parameters	Description	Data source	Modelled values
ITN effectiveness	Pyrethroid insecticide resistance	*d*_*N0*_,* r*_*N0*_, *H*_*py*_	Combination of 3 parameters for the probability of a mosquito being killed, probability of being repelled and ITN half-life. Entomological efficacy is conditional on pyrethroid resistance level and varies by ITN insecticide	Discriminatory dose susceptibility bioassays	20% increments between 0–100%
	Entomological efficacy of ITNs			Experimental hut trials and statistical modelling	10 parameter draws from fitted posterior distribution for each resistance level [7, 32]
Vector behaviour	Proportion of mosquito bites taken on humans (human blood index)	*Q*_*0*_	Vector behaviour influences the impact of vector control interventions and residual malaria transmission that can occur even with universal ITN and IRS use	Field studies on mosquito blood meals	74%, 92% [3]
	Proportion of mosquito bites taken indoors and proportion of bites taken in bed	${\Phi }_{I}$ and ${\Phi }_{B}$		Field studies using human landing catches or CDC light traps	78% and 69%, 97% and 90% [3]

All parameter draws were assumed to have equal probability and all uncertainty in identifying the optimal intervention was assumed to come from the four entomological indicators. Values for pyrethroid resistance and vector behaviour parameters were chosen to represent a uniform distribution between the indicated range. Rather than assigning these characteristics to individual vector species, uncertainty was represented in the average pyrethroid resistance and biting behaviour for all vectors participating in malaria transmission in the given setting

Monitoring of physiological insecticide resistance relies on standardized discriminatory dose bioassays, which quantify the proportion of mosquitoes that die after exposure to a discriminating concentration of insecticide [10]. The human blood index is measured by analysing mosquito blood meal sources, while data on indoor biting activity comes from human landing catches or CDC light traps together with surveys on human sleeping habits [29, 30]. These indicators are routinely monitored and often part of broader entomological surveillance studies, where mosquito collections also allow estimation of vector species composition, mosquito density and entomological inoculation rates [30]. Conversely, entomological efficacy of ITNs against mosquitoes is typically estimated using more complex and costly experimental hut trials [31]. Experimental huts allow to evaluate the effect of ITNs on blood-feeding and mortality of host-seeking mosquitoes under controlled conditions similar to those under which mosquitoes typically enter houses, following an assessment of susceptibility of the wild vector population to the active ingredient. Statistical models have been used to synthesize entomological trial data and local resistance profiles, and this relationship can be incorporated in mathematical models to project the potential impact of ITNs in various locations [7, 16]. In the model, ITNs are modelled probabilistically via their effect on the probability of a mosquito being killed, repelled from the ITN, or biting successfully. The entomological efficacy of the different classes of ITNs on these outcomes over time at different levels of pyrethroid insecticide resistance, as measured in bioassays, was previously estimated by fitting the model to experimental hut trial data [7, 32]. The analysis assumed no prior knowledge of pyrethroid insecticide resistance in the modelled generalized settings; this was varied uniformly between 0 and 100%. For entomological efficacy, 10 posterior parameter sets were randomly sampled conditional on the level of insecticide resistance to represent the uncertainty in ITN effectiveness (Table 2) (Appendix pp.20–22). For vector behaviour parameters, ranges for the proportion of bites taken on humans (74%–92%) [33] and for the proportion of bites taken indoors (78%–97%) or in bed (69%–90%) were based on the interquartile range in a systematic review of all common vector species in Africa [3].

### Cost-effectiveness and VOI analysis

All analyses were conducted in R and model simulations were performed using the *malariasimulation* package (v1.3.0) [34]. The modelled age-specific incidence of clinical and severe malaria for each scenario were used to derive the number of malaria-related deaths (assumed to represent 21.5% of severe cases) and disability-adjusted life years (DALYs) (Appendix p.23). Estimated costs for each intervention, derived from secondary data sources, are shown in Appendix pp.23–26, and were varied in sensitivity analyses. Costing of ITNs accounted for increasing marginal costs of ITN distribution as population coverage increases [35, 36]. Health economic methods are reported according to the CHEERS-VOI statement (Table S1.6) [37].

Firstly, to identify the optimal intervention package in each setting in the presence of uncertainty, the incremental cost and DALYs averted in each scenario compared to the baseline of maintaining existing interventions over 3 years were calculated. All intervention scenarios were compared to their corresponding baseline setting with existing ITN use, 45% treatment coverage, and, for highly seasonal settings, SMC at 85% coverage. From this, the net monetary benefit (NMB) of each intervention package *i* in each setting at different cost-effectiveness thresholds was derived according to the following equation:

$${\text{NMB}}_{i} =\, \left( {{\text{DALYs}}\;{\text{averted}}_{i} \times {\text{cost - effectiveness }}\;{\text{threshold}}} \right) - {\text{incremental}}\;{\text{cost}}_{i}$$

The NMB gives a measure of the value of an intervention in monetary terms at the given cost-effectiveness threshold. Cost-effectiveness thresholds represent the maximum amount a decision-maker would be willing to pay for an averted DALY. Estimates of health opportunity costs suggest values of less than 1 times the GDP per capita in almost all low- and middle-income countries [38]. Based on these estimates (Appendix p.23), the NMB was calculated at cost-effectiveness thresholds between US$75 and US$1000 and results are presented at thresholds of US$75, US$250, US$500 and US$1000, summarized as the median and 95% credible interval (CrI, 2.5th and 97.5th percentile). The optimal intervention package was defined as the package with the highest median net monetary benefit.

Secondly, to quantify the decision uncertainty in the choice of optimal intervention and estimate the potential value of data collection for all four entomological indicators, the expected value of perfect information (EVPI) was calculated in each setting as the difference between the expected value of an intervention package under certainty and the expected value under uncertainty, as implemented in the *voi* R package (v1.0.2) [39]:

$$EVPI = EV_{certainty} - EV_{uncertainty} = {\mathbb{E}}_{j} \left[ {{\text{max}}_{i} V\left( {i,j} \right)} \right] - max_{i} {\mathbb{E}}_{j} \left[ {V\left( {i,j} \right)} \right]$$

*V*(*i,j*) refers to the NMB of intervention package *i* under parameter set *j*. The expected value ${\mathbb{E}}_{j}\left[{\text{max}}_{i}V\left(i,j\right)\right]$ represents the mean of the highest NMB for each parameter set *j*. The expected value ${\mathbb{E}}_{j}[V\left(i,j\right)]$ is the mean NMB of each intervention package *i* across parameter sets *j*, where max_*i*_ indicates that the intervention with the highest expected value is chosen under uncertainty. A worked example of calculating the EVPI in terms of the opportunity cost associated with a decision is shown in Panel A [40, 41]. The population at risk of malaria in the area in which the intervention package is implemented would benefit from reduced decision uncertainty. The EVPI was therefore calculated per person at risk, and summarized as the median, interquartile range (IQR) and range across settings. The EVPI can be interpreted as the theoretical maximum that a decision-maker should be willing to invest into data collection on the entomological parameters for a decision on these specific interventions.

The expected value of partial perfect information (EVPPI) was also calculated on the two groups of parameters governing ITN effectiveness and vector behaviour in the model (Table 2). The EVPPI represents the value of eliminating uncertainty in a subset of parameters while the other parameters remain uncertain. It was calculated using the Gaussian process regression method in the *voi* package [42]. Model fits were assessed by plotting the residuals against fitted EVPPI values.

### Analysis of funding for entomological monitoring

The US President’s Malaria Initiative (PMI) is the largest funder of entomological monitoring for malaria [43]. To estimate current PMI investments into entomological data collection in Africa, budget data for 2021–2023 was extracted from annual country-specific malaria operational plan funding tables on the PMI website [44]. Funding for the “Support Entomological Monitoring” and “Support ITN Durability Monitoring” categories were included. The total budget for the 3-year period was divided by the population at risk of malaria in each country to compare investments per person at risk with estimates of the VOI.

Panel A: Cost-effectiveness and value of information analysis: definitions and examples **Cost-effectiveness threshold**

Represents the maximum amount a decision-maker would be willing to pay for an averted DALY, which depends on the setting. Cost-effectiveness thresholds represent health opportunity costs of investment in a new intervention, i.e. the improvement in health that would have been possible if these resources were instead invested in other healthcare activities

**Net monetary benefit**

The net monetary benefit (NMB) of an intervention is an outcome measure of its value for money and combines information about its effectiveness and costs relative to current practice. It is calculated as:

$$NMB = \left( {DALYs\;averted \times cost\hbox{-}effectiveness\;threshold} \right) - incremental\;cost$$

As illustrated in the following diagram, the intervention with the highest net monetary benefit represents the most cost-effective option at a given cost-effectiveness threshold. Below the black horizontal line, neither intervention would be cost-effective (NMB < 0) and both would be dominated by current practice.
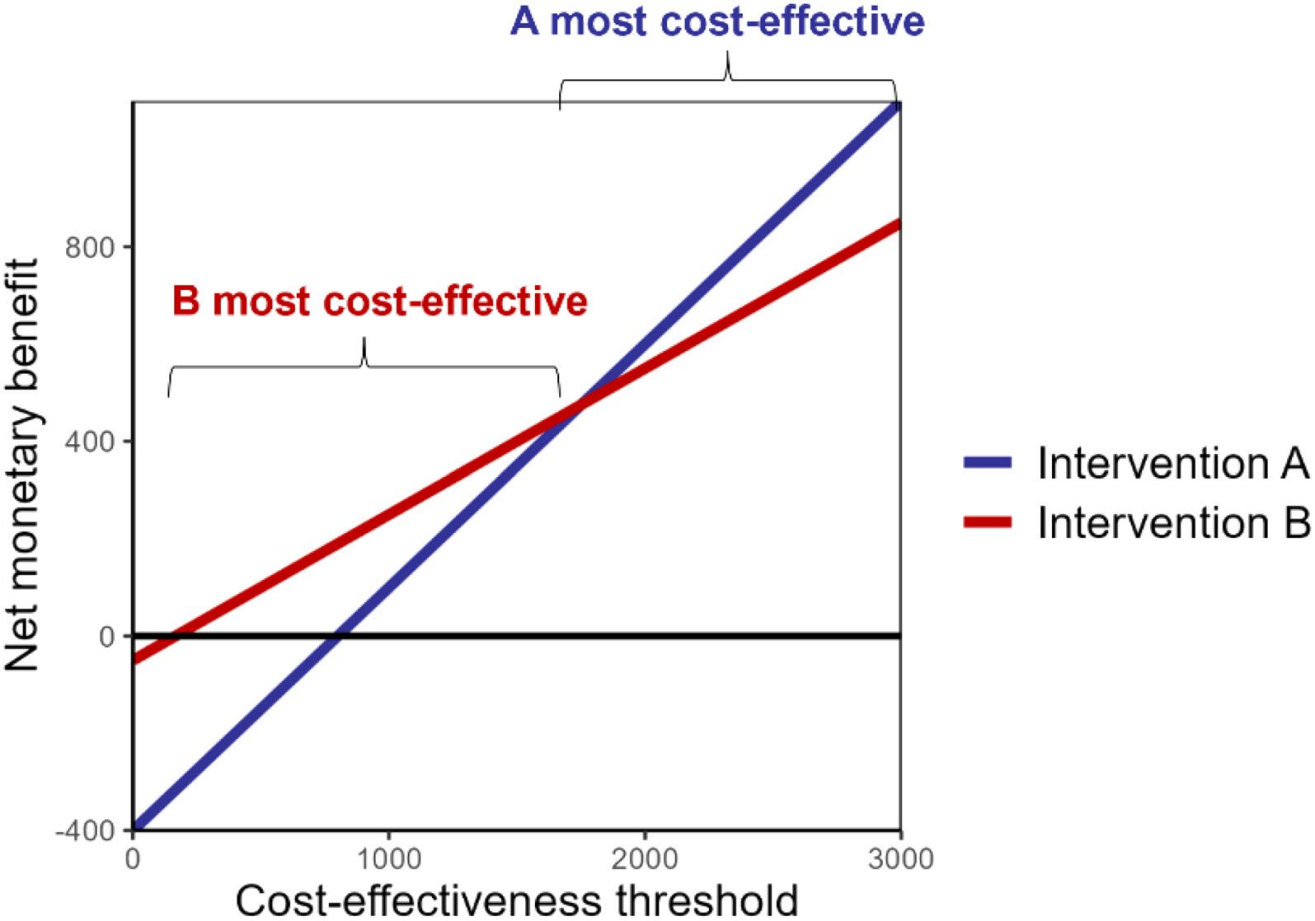

**Value of information analysis**

Enables estimation of the value of eliminating uncertainty through further data collection before making a decision on intervention selection. The value of information is obtained by quantifying the probability and magnitude of the potential opportunity cost associated with a decision. The value of information represents the monetary value of removing uncertainty in all (expected value of perfect information, EVPI) or a subset (expected value of partial perfect information, EVPPI) of the model parameters that influence the optimal intervention choice. It can be interpreted as the theoretical maximum amount that might be worth investing into data collection to reduce uncertainty in these parameters.

**Expected value of perfect information**

Calculation of the EVPI is illustrated in the following hypothetical example, where the net monetary benefit (NMB) of two interventions (or two intervention packages), A and B, is compared to current practice. The expected value of perfect information in deciding between these three interventions is calculated, assuming that uncertainty in their NMB arises from only three different parameter sets (rows) with equal probability. The EVPI quantifies the uncertainty in which intervention has the higher NMB.
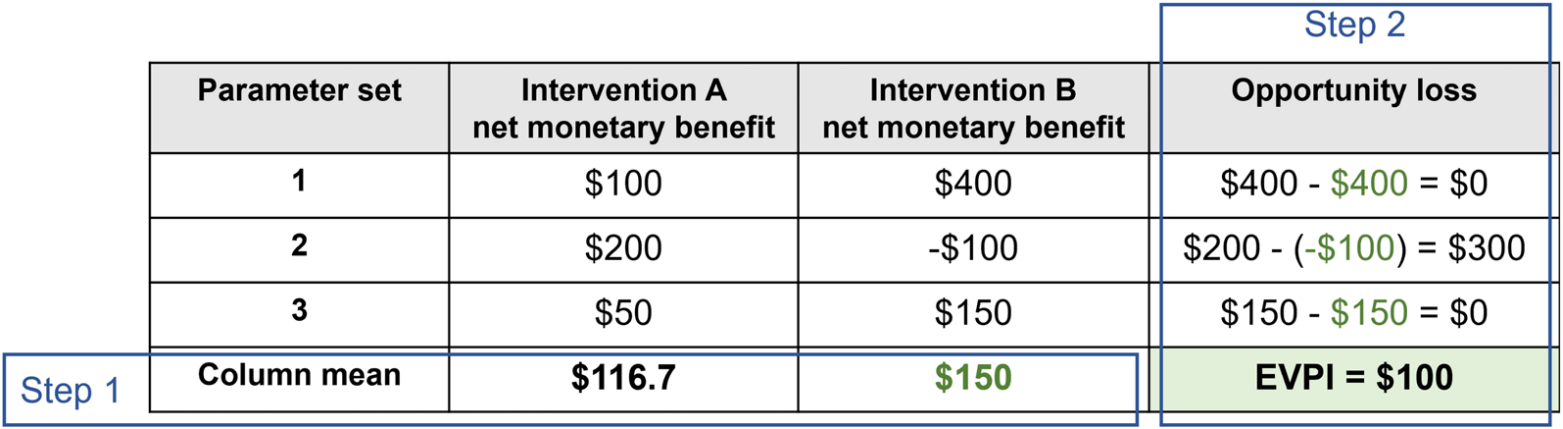

Model outputs consist of the NMB of each intervention for each parameter set. The first step in the value of information analysis is to calculate the mean NMB of each intervention across parameter sets. Both interventions are on average cost-effective compared to current practice (NMB > 0). In the presence of uncertainty, intervention B is considered to be the most cost-effective based on having the highest mean NMB ($150 vs $116.7). The second step is to calculate the potential opportunity cost for each parameter set when choosing the most cost-effective intervention, B. This involves subtracting, in each row, the NMB of intervention B from the highest NMB. For parameter sets where intervention B has the highest NMB, having selected this intervention incurs no opportunity cost ($0). The EVPI is defined as the mean opportunity costs across parameter sets, which in this decision problem equals ($0 + $300 + $0)/3 = $100. This EVPI for the varied parameters represents a sizable proportion, $100/$150 * 100 = 67%, of the expected value (NMB) of immediately implementing the optimal intervention, B.

## Results

### Cost-effectiveness analysis

Across the range of settings explored, the modelled intervention packages without IRS (case A) averted between 365 (95% CrI − 48–1050) and 3,581 (95% CrI 1774–8854) cases per 10,000 persons at risk of malaria over a 3-year period compared to existing interventions at baseline (Fig. S2.2). The incremental costs of their implementation were estimated at between US$3,496 (95% CrI 1903–5201) and US$83,890 (95% CrI 19,272–116,650) per 10,000 persons at risk. These costs were partially compensated by savings due to reduced treatment need of between US$1,372 (95% CrI − 377–4523) and US$19,922 (95% CrI 10,332–41,395) per 10,000 persons at risk.

**Fig. 2:**
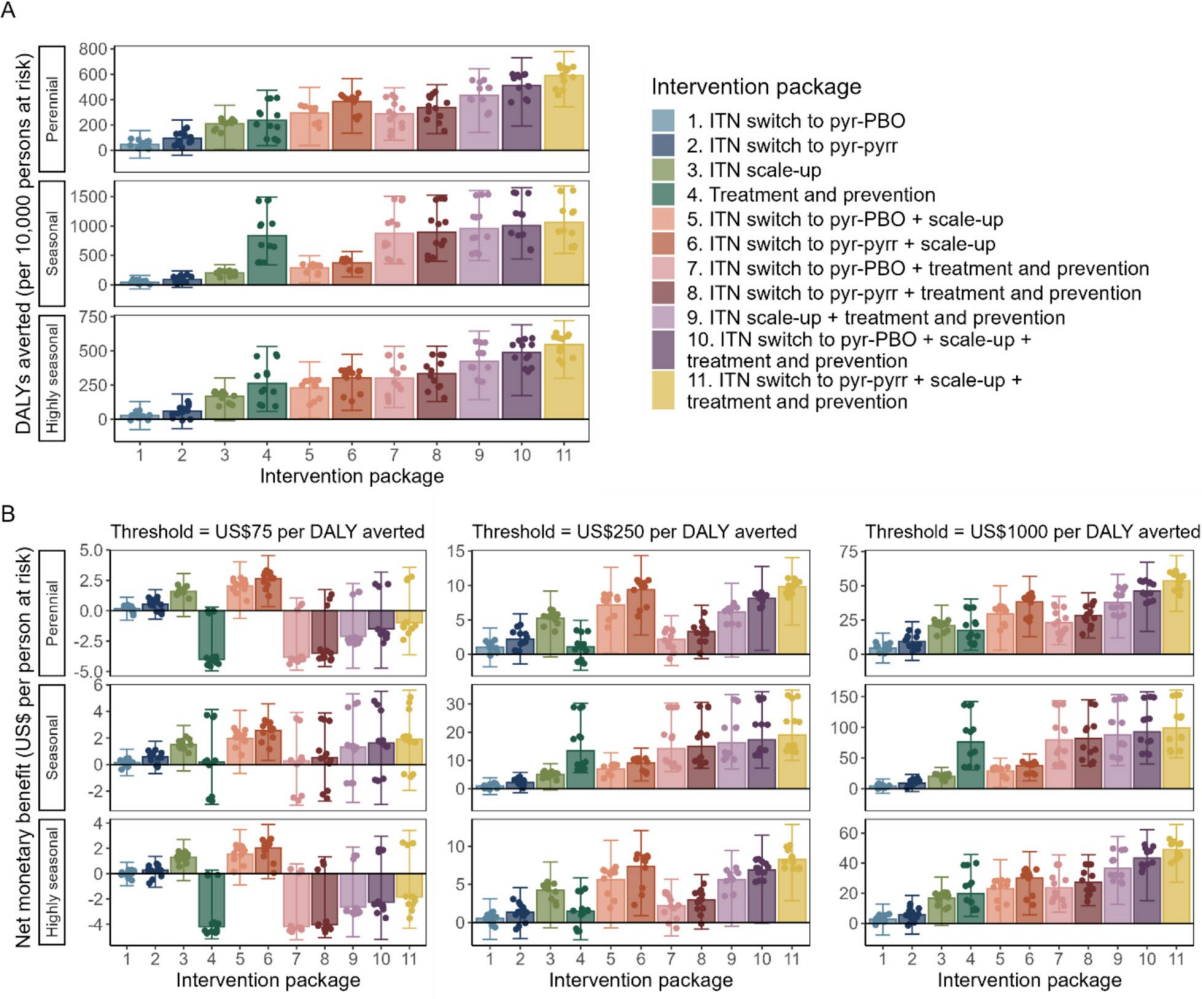
Impact and cost-effectiveness of the different intervention packages relative to the corresponding baseline setting with existing interventions, stratified by seasonality. Bars represent the median across settings (including *Pf*PR_2–10_ 5% to 40% and ITN use 20% to 60%), with error bars indicating the 95% credible interval (across settings and parameter variation). Points represent the median value in each prevalence and baseline ITN use setting (12 for each intervention per seasonality panel). The “treatment and prevention” package consists of a combination of treatment scale-up, SMC and/or RTS,S vaccination depending on the setting. **A** DALYs averted by the different intervention packages over 3 years. In seasonal settings, intervention packages including treatment and prevention avert more DALYs than in other seasonality settings because of the inclusion of SMC (not applicable in perennial settings and assumed to be already implemented at baseline in highly seasonal settings). **B** Net monetary benefit (NMB) of the different intervention packages over 3 years at cost-effectiveness thresholds of US$75, US$250 and US$1000 per DALY averted. A positive NMB indicates that the intervention is cost-effective compared to existing interventions at baseline; the optimal intervention package is the one with the highest average NMB. Calculation of the NMB depends on assumptions about the value of an averted DALY, therefore NMBs are higher for a cost-effectiveness threshold of US$1000 per DALY averted than the US$250 threshold

Figure 2A shows the DALYs averted by the intervention packages compared to existing interventions at baseline in perennial, seasonal and highly seasonal settings, which was highest for the combination of switching to pyrethroid-pyrrole ITNs, scaling-up ITN usage and introducing the treatment and prevention package. The net monetary benefit considers the DALYs averted as well as the incremental costs of an intervention package, with the highest average NMB representing the optimal intervention package at a specific cost-effectiveness threshold (Fig. 2B). For cost-effectiveness thresholds ≥ US$150 per DALY averted, all interventions were likely to be cost-effective compared to existing interventions at baseline (median NMB > 0). Without IRS, switching to pyrethroid-pyrrole ITNs with ITN scale-up represented the optimal intervention package across settings at the threshold of US$75 per DALY averted, while intervention packages including the treatment and prevention package were not cost-effective in perennial and highly seasonal settings. At higher cost-effectiveness thresholds (≥ US$170), switching to pyrethroid-pyrrole ITNs with ITN scale-up and the treatment and prevention package was the most cost-effective intervention package across settings, with a NMB of US$10.6 (95% CrI 4.0–33.6) and US$56.9 (95% CrI 31.0–156.0) per person at risk at cost-effectiveness thresholds of US$250 and US$1000 per DALY averted, respectively.

NMBs varied across transmission settings (Fig. 2B), but “ITN switch to pyrethroid-pyrrole + ITN scale-up + treatment and prevention” had the highest probability of being the most cost-effective intervention across settings (47–87%) for cost-effectiveness thresholds between US$170 and US$1000. This intervention package also had the highest probability of being most cost-effective in 81% and 100% of 36 transmission settings at cost-effectiveness thresholds of US$250 and US$1000, respectively (Fig. S2.3). However, there was uncertainty about the optimal intervention package in each transmission setting: the most common optimal alternatives to “ITN switch to pyrethroid-pyrrole + ITN scale-up” were “ITN switch to pyrethroid-pyrrole + ITN scale-up + treatment and prevention” and “ITN switch to pyrethroid-PBO + ITN scale-up”, and the most common optimal alternatives to “ITN switch to pyrethroid-pyrrole + ITN scale-up + treatment and prevention” were “ITN switch to pyrethroid-PBO + ITN scale-up + treatment and prevention” and “ITN switch to pyrethroid-pyrrole + ITN scale-up”. The optimal intervention package included changing from a pyrethroid-only ITN in at least 93% of cases.

Intervention packages including IRS (case B) had considerably higher impact [11,033 (95% CrI 4256–21,825) cases averted per 10,000 for IRS alone] but were also much more expensive than other interventions [incremental cost of US$137,456 (95% CrI 97,401–167,438) per 10,000 for IRS alone after accounting for cost savings] (Fig. S2.4). IRS accounted for 70% (95% CrI 59–98) of total incremental programme costs of the “ITN switch to pyrethroid-pyrrole + treatment and prevention + IRS” intervention package. IRS was not cost-effective across settings at the cost-effectiveness threshold of US$75, but had the highest probability of being the most cost-effective intervention at cost-effectiveness thresholds of US$250 and US$500 (53% and 38%, respectively). At higher cost-effectiveness thresholds, switching to pyrethroid-pyrrole ITNs together with IRS had the highest probability (38% at US$1000 per DALY averted). Overall there was more decision uncertainty when considering intervention packages including IRS (Fig. S2.5).

### VOI analysis

Table 3 shows the expected value of perfect information on the entomological indicators for cost-effectiveness thresholds between US$75 and US$1000 per DALY averted. When considering all interventions except for IRS (case A), the median EVPI associated with resolving uncertainty in all entomological indicators ranged from US$0.05 (IQR 0.03–0.08) to US$0.17 (IQR 0.12–0.43) per person at risk at cost-effectiveness thresholds of US$75 and US$1000, respectively. On average, this represented 0.3–1.8% of the benefits achieved through immediate implementation of the optimal intervention package (Table 3). The EVPI on entomological indicators was similar if IRS was considered as a possible intervention (case B). When varying assumptions on intervention costs in sensitivity analyses, EVPI estimates were most sensitive to the relative costs of pyrethroid-PBO and pyrethroid-pyrrole ITNs (Appendix pp.35–36). Decision uncertainty was significantly reduced if the cost of pyrethroid-pyrrole ITNs was halved while other costs stayed constant, but increased if the cost of pyrethroid-PBO ITNs was further reduced compared to that of pyrethroid-pyrrole ITNs.

**Table 3:** Expected value of perfect information on entomological indicators

Cost-effectiveness threshold	Expected value of perfect information			
	Absolute value (US$ per person at risk)		Relative value (percentage of net monetary benefit provided by optimal intervention package)	
	Median [IQR]	Range	Median [IQR]	Range
Case A: all interventions excluding IRS				
75	$0.05 [$0.03–$0.08]	$0.02–$0.23	1.8% [0.9%–3.5%]	0.5%–30.6%
250	$0.07 [$0.05–$0.19]	$0.03–$0.53	0.5% [0.4%–2.1%]	0.2%–7.6%
500	$0.12 [$0.08–$0.24]	$0.05–$0.80	0.4% [0.3%–0.9%]	0.1%–3.7%
1000	$0.17 [$0.12–$0.43]	$0.09–$1.43	0.3% [0.2%–0.5%]	0.1%–2.7%
Case B: all interventions including IRS				
75	$0.03 [$0.01–$0.05]	$0.00–$0.29	1.0% [0.1%–2.0%]	0.0%–10.9%
250	$0.06 [$0.02–$0.15]	$0.00–$0.41	0.2% [0.1%–0.3%]	0.0%–3.8%
500	$0.15 [$0.09–$0.19]	$0.03–$0.42	0.2% [0.2%–0.3%]	0.1%–0.4%
1000	$0.28 [$0.25–$0.32]	$0.20–$0.75	0.2% [0.2%–0.3%]	0.1%–0.4%

The expected value of perfect information applies to a 3-year period, is summarized across settings, and expressed in 2023 US dollars per person at risk and relative to the net monetary benefit provided by the optimal intervention package in each setting. Cost-effectiveness thresholds are expressed in US dollars per DALY averted. Assumptions on entomological parameter uncertainty are detailed in Table 2

*IQR* interquartile range

**Fig. 3:**
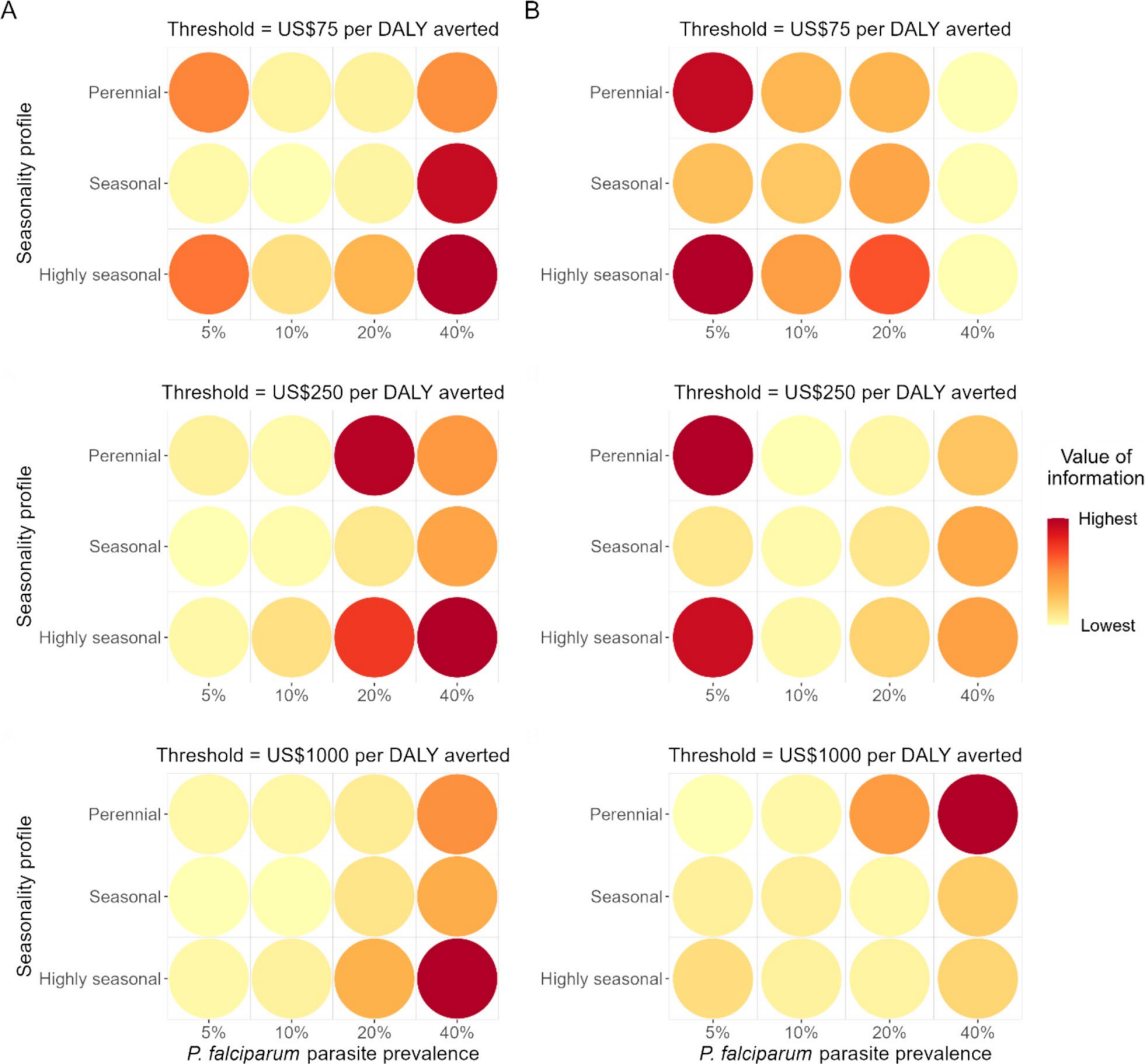
Expected value of perfect information (EVPI) by prevalence and seasonality setting for the set of interventions **A** without IRS and **B** including IRS. The EVPI is shown for cost-effectiveness thresholds of US$75, US$250 and US$1000 per DALY averted and averaged across ITN use levels in each setting

The EVPI varied widely across settings, with estimates as high as US$0.23 (30.6% of intervention net benefits) and US$1.43 per person at risk (2.7% of intervention net benefits) at cost-effectiveness thresholds of US$75 and US$1000, respectively. Where the VOI was highest depended on the cost-effectiveness threshold and the interventions under consideration. Without IRS, the settings with the highest VOI estimates had a parasite prevalence of 20% or 40% and highly seasonal transmission (Fig. 3A). At a cost-effectiveness threshold of US$75 per DALY averted, the VOI was also high in the 5% prevalence setting with perennial or highly seasonal transmission. At higher cost-effectiveness thresholds, high-prevalence settings also had the highest VOI when IRS was considered, but mainly with perennial transmission (Fig. 3B). However, this was not the case at lower cost-effectiveness thresholds; the VOI was lower in high-prevalence settings because decision uncertainty was reduced through intervention packages containing IRS having a substantially higher net monetary benefit than other interventions. With IRS and at a cost-effectiveness threshold of US$75 and US$250, the EVPI was highest in perennial and highly seasonal settings with 5% prevalence. At the US$75 threshold, the EVPI was lowest in the 40% prevalence setting. In all other cases with and without IRS, the EVPI was lowest in settings with a lower parasite prevalence of 10%. Baseline ITN use did not strongly affect estimates.

**Fig. 4:**
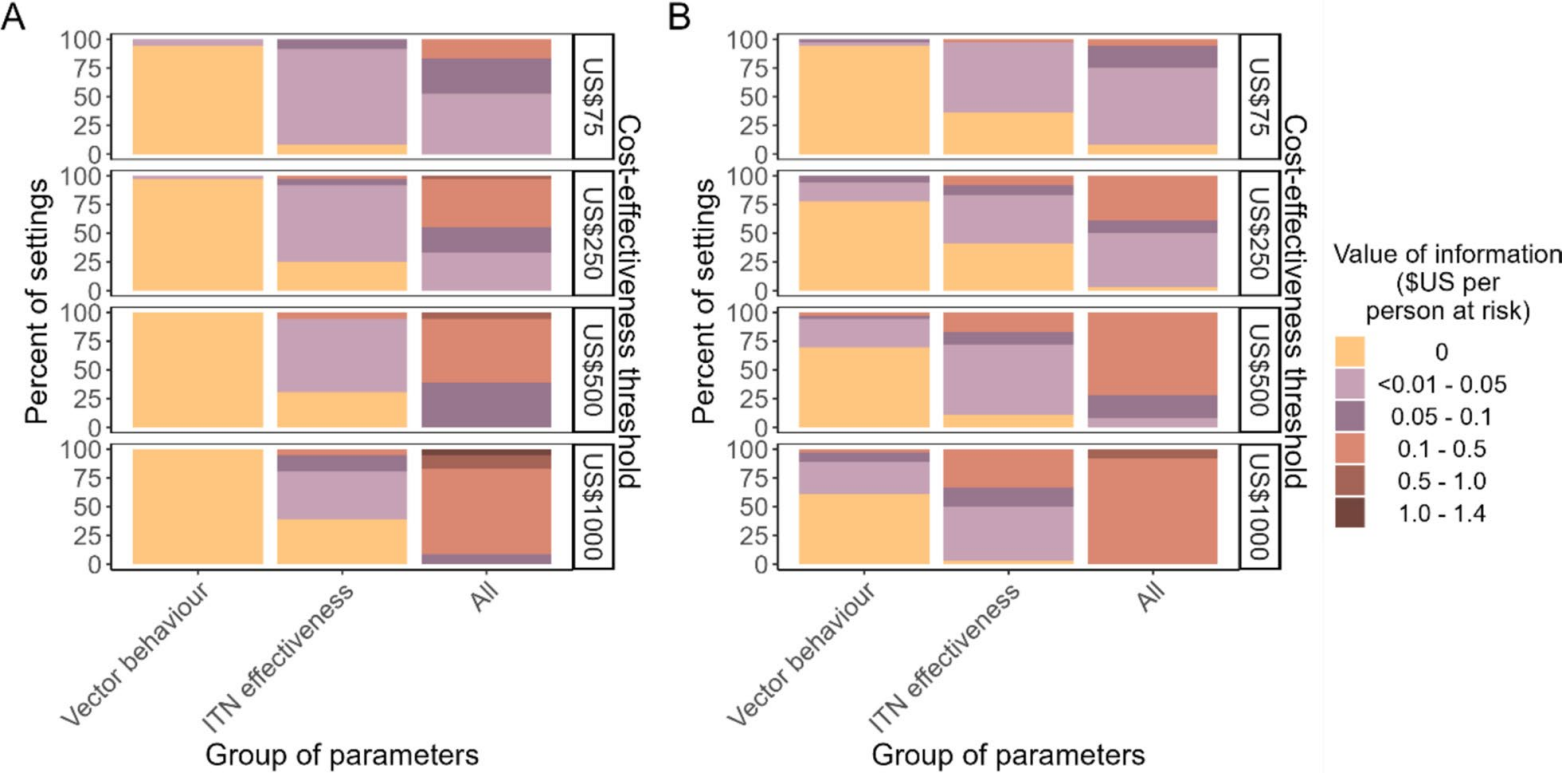
Value of information on different groups of entomological indicators for the set of interventions **A** without IRS and **B** including IRS. The distribution of value of information estimates across settings is shown when eliminating uncertainty in pyrethroid resistance and ITN entomological efficacy combined (expected value of partial perfect information (EVPPI) on ITN effectiveness), in the proportion of mosquito bites taken on humans and taken indoors/in bed combined (EVPPI on vector behaviour parameters), and the expected value of perfect information (EVPI) on all four entomological indicators combined. The last column for all indicators corresponds to the EVPI values in Table 3

Figure 4 shows the expected value of partial perfect information on two subsets of the entomological indicators. Without IRS, the EVPPI on the vector behaviour parameter group was 0 in all but one setting (Fig. 4A), meaning that additional information on these parameters currently adds no benefit in selecting the optimal intervention**.** Including IRS, the EVPPI on vector behaviour was comparatively higher even with remaining uncertainty in the resistance-dependent entomological efficacy of ITNs (Fig. 4B). With and without IRS, the EVPPI was higher for the setting-specific ITN effectiveness, represented through combined elimination of uncertainty in pyrethroid resistance and the corresponding entomological efficacy of ITNs. The median EVPPI was US$0.009 (IQR 0.003–0.017) and US$0.011 (IQR 0.000–0.031) per person at risk at cost-effectiveness thresholds of US$75 and US$1000, respectively, without IRS, compared to US$0.003 (IQR 0.000–0.010) and US$0.048 (IQR 0.023–0.112) with IRS. Nevertheless, in settings where the EVPPI was greater than 0, the VOI on all indicators (EVPI) across settings was on average 4–41 times higher than the EVPPI, meaning that knowledge about all four indicators was more valuable to a decision-maker than information about vector behaviour or ITN effectiveness alone.

**Fig. 5:**
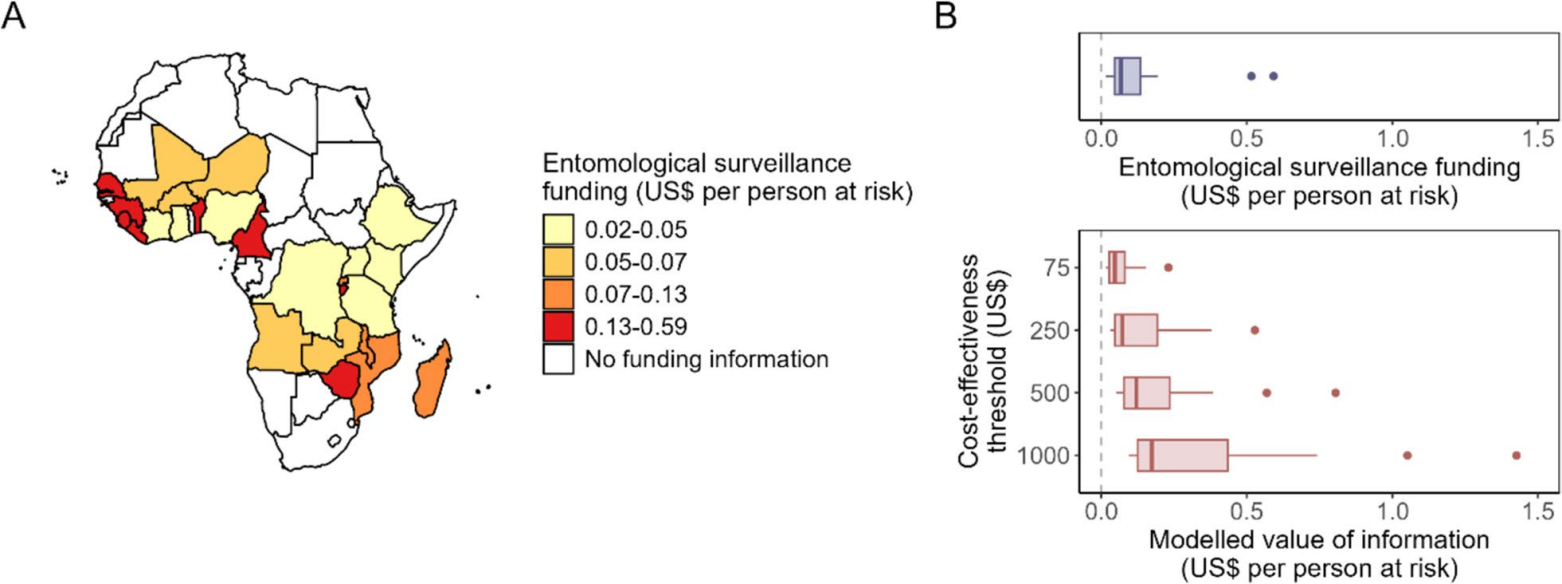
Comparison of the estimated expected value of perfect information (EVPI) with investments into entomological monitoring by the US President’s Malaria Initiative (PMI) in 2021–2023. **A** Distribution of PMI entomological surveillance funding across African countries. Investments in US dollars were divided by the population at risk of malaria in the respective country, with funding bands representing quartiles. **B** Comparison of these investments with the estimated EVPI for the set of interventions without IRS as shown in Table 3. Countries without PMI funding were excluded from this comparison

Estimates of the VOI can be interpreted as a theoretical maximum that might be worth investing into data collection on these entomological indicators, assuming that further data collection can provide the required information. The estimated EVPI was compared to data on current investments into entomological monitoring from the US President’s Malaria Initiative for the 2021–2023 period on the country level, with Fig. 5A showing the distribution of funding per population at risk across countries in sub-Saharan Africa. Twenty-five countries were supported by PMI and received funding for entomological monitoring. Among those, the median funding per person at risk for the 3-year period was US$0.07 (IQR 0.05–0.13, range 0.02–0.59). This corresponded very closely to the estimated VOI for all entomological indicators at the cost-effectiveness threshold of US$250 per DALY averted, while the VOI was higher at the higher cost-effectiveness thresholds (Fig. 5B).

## Discussion

This study assessed the most cost-effective malaria intervention package and quantified the potential monetary value of entomological data collection for programmatic decision-making in different sub-Saharan African settings. An intervention package involving a switch to pyrethroid-pyrrole ITNs, scaling up ITNs and scaling up other treatment and prevention interventions represented the optimal combination on average at cost-effectiveness thresholds over US$170 where IRS is not considered. In all settings there was value to obtaining further information, with a median expected value of perfect information (EVPI) of US$0.05–US$0.17 per person at risk depending on the cost-effectiveness threshold. The EVPI varied across settings, but in most the decision on the optimal intervention could be made based on existing evidence as the VOI was modest compared to the net monetary benefit of its immediate implementation. However, the VOI is potentially still higher than current PMI funding for entomological monitoring in many settings and increased investments may, therefore, be warranted.

These results provide further support for the cost-effectiveness of new pyrethroid-pyrrole ITNs across a variety of malaria transmission settings [14, 32], despite a relatively smaller projected impact on averted DALYs than on averted clinical cases. This was due to a rebound in severe cases occurring in high-prevalence settings under some parameter assumptions over the time horizon considered. In children protected by highly effective new ITNs in the first year after distribution, a delay in immunity development led to an increase in severe cases in the third year in the model, when ITN efficacy had partially waned and usage had dropped. This is similar to observed rebound patterns after malaria vaccination and other interventions [45], but unlikely to occur in reality where ITNs are also distributed through routine mechanisms in addition to the mass campaigns modelled here [46]. Compared to previous studies, this study also shows that switching to new nets could be cost-effective as part of a comprehensive intervention package, even if there is uncertainty about the exact pyrethroid resistance level and the entomological efficacy of different classes of nets in a setting. Comparison of the estimated VOI to other studies is challenging because of methodological differences. The EVPI on different entomological parameters in a previous study on decision-making for IRS and larviciding interventions in East Africa was higher than the estimates in this study [23], but this was partly due to a much higher cost-effectiveness threshold (US$7773).

Without IRS, estimates of the VOI were considerably higher in the highest prevalence (40%) setting (particularly with high seasonality) than in others, suggesting that entomological data collection could be prioritized geographically. The VOI was in many cases lower in seasonal than in perennial and highly seasonal settings due to the inclusion of highly (cost-)effective SMC, which lowered the decision uncertainty in including the treatment and prevention package. However, these results were highly dependent on the interventions under consideration and the assumed cost-effectiveness thresholds, and so it was not possible to draw a general conclusion on which settings to prioritize for entomological data collection overall. Concomitantly, eliminating uncertainty in all entomological indicators had significantly higher value than only eliminating uncertainty in the subsets of parameters related to ITN effectiveness or to vector behaviour. ITN effectiveness parameters contributed more to the overall EVPI because much decision uncertainty arose from the choice of different net classes, while vector behaviour became more relevant if IRS was also considered. The EVPPI on vector behaviour was lower both because these parameters were assumed to be less uncertain and because they had little effect on differences in the net monetary benefit between intervention packages. Overall, the results therefore support integrated programmatic surveillance platforms collecting data on a range of entomological factors over studies on individual indicators [8]. Whether obtaining this additional evidence is worthwhile ultimately depends on the relationship between the VOI and the cost of data collection and how accurate it is. However, it was not possible to identify sufficient data to estimate if the cost of surveillance activities able to generate data on the entomological indicators in the model would be less than the estimated VOI. In practice, data collection on the entomological indicators can also only provide imperfect information (which is why the EVPI represents a potential maximum value). Susceptibility bioassays to determine resistance levels are associated with large measurement error, particularly in high-resistance areas [2, 7], and the variability in experimental hut trials and mosquito bionomics studies is also substantial [3, 33, 47].

In interpreting the results, it should be kept in mind that the aim was to estimate the VOI for a specific decision problem across generalized settings in sub-Saharan Africa. The results show that the VOI depends on the specific intervention packages under consideration. Currently, the most widely used vector control tools (ITNs and IRS) target the mosquito indoors, so information on the level of residual transmission is less informative. This will likely change if other interventions become available, making entomological information more valuable. Conversely, the sensitivity analysis on intervention costs shows that the value of information would be reduced if the cost of pyrethroid-pyrrole ITNs was lower than currently estimated relative to pyrethroid-PBO ITNs. The analysis also focuses on a cross-sectional assessment of the VOI for a 3-year decision on an intervention package, so estimates do not directly apply to the value of continuous monitoring and longitudinal data collection in sentinel sites [8]. The value of entomological data collection is likely to change over time, particularly with changes to control strategies, and similar analyses could be conducted to inform withdrawal of vector control interventions as transmission decreases. Importantly, entomological surveillance has additional uses beyond the decision problem in this study; examples include the targeting and optimal allocation of resources across locations, providing knowledge about the extent and trends in insecticide resistance to inform resistance management strategies, and awareness of emerging threats like the spread of *Anopheles stephensi* [9]. The EVPI estimates presented here should therefore not be interpreted as a measure of how much to invest in entomological surveillance systems overall without considering these additional factors.

There are several limitations to this work. Firstly, to ensure computational feasibility, uncertainty was only represented for the four key entomological parameters known to influence the impact of vector control interventions in model simulations and for which further data can be collected with existing entomological methods [8]. The assumption of no uncertainty in other aspects of local epidemiology, e.g. parasite prevalence and seasonality, or in the underlying mosquito biology and life cycle, may have underestimated the overall EVPI compared to the real-life knowledge in a setting [8]. Similarly, the point estimates used do not fully capture the uncertainty distribution of the vector behaviour parameters. Secondly, results apply to generalized settings and uncertainty ranges for the entomological indicators were derived across sub-Saharan Africa. However, in a given setting, additional prior knowledge about the parameters may exist due to past data collection, and thus the EVPI may be reduced. It was also assumed that entomological parameters would remain the same over the course of the 3-year simulation period. While this work has explored a wide range of plausible scenarios for Africa, the considerable variability in the VOI between settings according to historical knowledge and local epidemiological and entomological factors means that more tailored analysis is recommended. Decision-making should ideally be based on country-specific or subnational analyses using local data, for example using the MINT online tool which has been piloted with the Ghana National Malaria Elimination Programme [7, 48]. Thirdly, the comparison of the estimated VOI with current investments into entomological monitoring should be interpreted with caution. Data on these were only available from one funder, and no information was available for large parts of sub-Saharan Africa. Model outputs are not representative of the distribution of transmission settings and associated population at risk across countries in sub-Saharan Africa, which hinders a direct comparison with country-level data. Fourthly, these results may overestimate the impact and cost-effectiveness of IRS, as the model does not account for practical challenges that limit the effective coverage of spraying campaigns, such as mistiming with regards to the rainy season and household modifications after application [49].

## Conclusion

This study suggests that entomological data collection in the short-term should not delay implementation of interventions that have been empirically tested, have demonstrated efficacy and have been recommended by the WHO. However, the value of information on entomological indicators is likely to exceed current PMI funding for entomological monitoring in many settings. Sustained investments into entomological surveillance are, therefore, worthwhile and have broad value to national malaria programmes.

## Supplementary Information

Supplementary Material 1.

## Data Availability

No datasets were generated or analysed during the current study.
