# Supplementary material for: Quantifying the potential value of entomological data collection for programmatic decision-making on malaria control in sub-Saharan African settings
Source: Malar J. 2025 Jan 30;24:31. doi: 10.1186/s12936-025-05251-7 (PMC11780794; doi:10.1186/s12936-025-05251-7)
Supplement: Supplementary file 1 — Supplementary Material 1. [file 12936_2025_5251_MOESM1_ESM.docx]

**Supplementary Appendix**

# Quantifying the potential value of entomological data collection for programmatic decision-making on malaria control in sub-Saharan African settings

Nora Schmit^1^, Hillary M Topazian^1^, Matteo Pianella^1,2^, Giovanni D Charles^1^, Peter Winskill^1^, Penelope A Hancock^1^, Ellie Sherrard-Smith^1,3^, Katharina Hauck^4^, Thomas S Churcher^1^, Azra C Ghani^1^

1 MRC Centre for Global Infectious Disease Analysis, Imperial College London, London, United Kingdom

2 Department of Economics, Stockholm University, Stockholm, Sweden

3 Liverpool School of Tropical Medicine, Liverpool, United Kingdom

4 MRC Centre for Global Infectious Disease Analysis, Jameel Institute, Imperial College London, London, United Kingdom

Table of Contents

[1. Supplementary methods 3](#_Toc185433676)

[Transmission model 3](#_Toc185433677)

[Human component 3](#_Toc185433678)

[Model parameters 8](#_Toc185433679)

[Vector component 11](#_Toc185433680)

[Interventions 14](#_Toc185433681)

[Baseline settings and intervention schedules 19](#_Toc185433682)

[Parameter sets for entomological efficacy of ITNs 20](#_Toc185433683)

[Calculation of DALYs 23](#_Toc185433684)

[Costing and cost-effectiveness thresholds 23](#_Toc185433685)

[CHEERS-VOI reporting checklist 27](#_Toc185433686)

[2. Supplementary results 31](#_Toc185433687)

[Sensitivity analysis on costs 35](#_Toc185433688)

[3. References 37](#_Toc185433689)

# Supplementary methods

## Transmission model

### Human component

Individuals in *malariasimulation* enter the model at birth as susceptible to infection $(S)$ and with a level of maternal immunity that exponentially decays during the first 6 months of age (**Figure S1.1, Table S1.1**). Throughout the life course, individuals are exposed to infectious mosquito bites with the hazard of infection dependent on the force of infection from mosquito to human $(\Lambda_{i})$. This force of infection $\Lambda_{i}$depends on an individual’s level of pre-erythrocytic immunity and vector parameters such as population size, biting rate, and infectiousness. Upon infection, individuals experience a 12-day latent period $(d_{E})$ before developing asymptomatic infection $A$ or clinical disease $D$ with a probability $(\phi_{i})$ dependent on an individual’s existing level of immunity against clinical disease. Diseased individuals are treated with probability $f_{T}$; treated individuals move to infection state $T$ and non-treated individuals move to diseased state $D$. Treated individuals recover from infection at rate $r_{T}$ and acquire prophylactic protection from re-infection; this protection wanes over time until individuals return to the susceptible state $S.$Untreated individuals move from $D$ to $A$ at rate $r_{D}$, then from $A$ to a sub-patent infection state $U$ with rate $r_{A}$ as parasite density declines, then from $U$ to $S$ at rate $r_{U}$. The model allows for super-infection (re-infection) of individuals in states $D$, $A$ and $U$.

The total population size remains constant across the entire simulation, as all-cause deaths are replaced with births of new individuals with the same individual biting rates. All simulations were performed in a population of 200,000, with the population age structure and corresponding death rates derived from a life table from Tanzania (1). Malaria-specific deaths are not included in the model itself but calculated from model outputs.


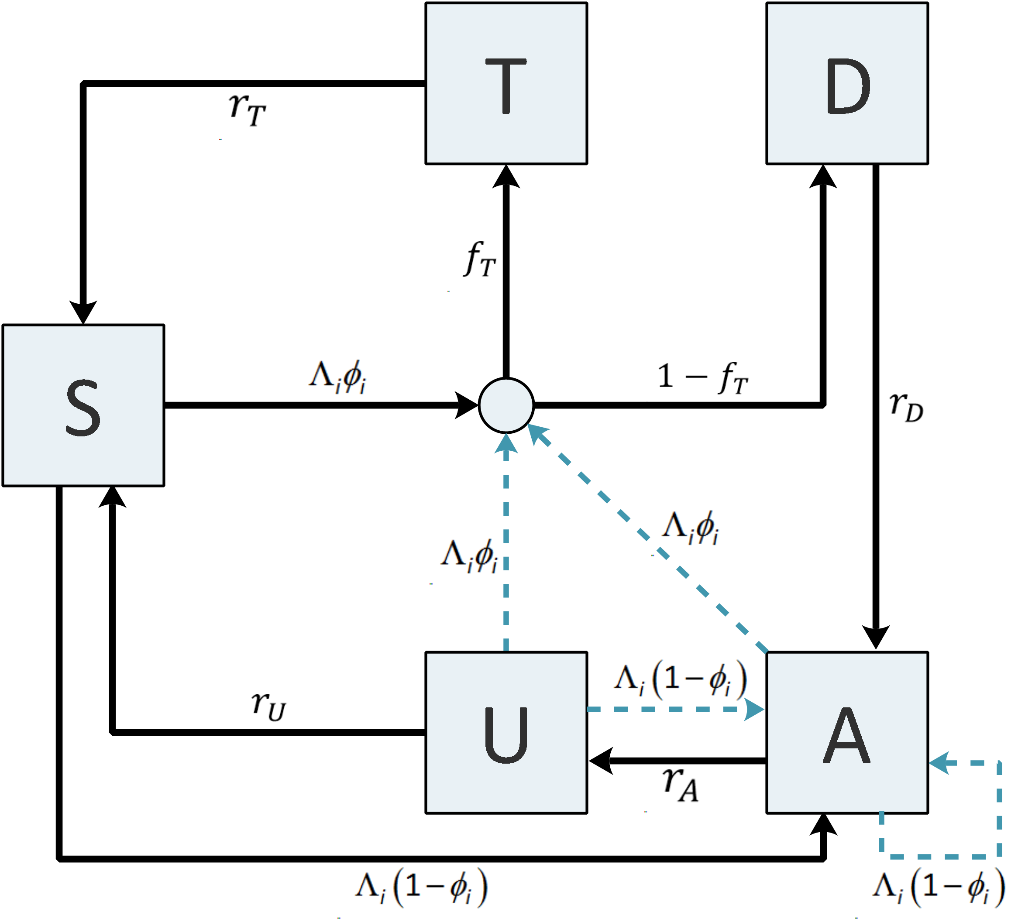


**Figure S1.1**. Flow diagram for the human transmission model, previously published by Winskill et al. 2017 (2). States are shown in boxes and transitions are shown via arrows; dashed arrows represent superinfection. Hazard rates are shown above transition arrows. Note: S = susceptible; T = treated clinical disease; D = untreated clinical disease; A = asymptomatic patent infection; U = asymptomatic sub-patent infection. All parameters and rates are described and referenced within **Table S1**.

**Table S1.1.** Infection state transition rates between human compartments. The overall infection process is shown as 🡪I for clarity, but involves progression from the susceptible state to either of compartments A, D or T.

| **Process** | **Transition** | **Rate** |
| --- | --- | --- |
| Infection (overall)  Development of asymptomatic infection  Development of untreated clinical disease  Development of treated clinical disease | S 🡪 I  S 🡪 A  S 🡪 D  S 🡪 T | $\Lambda_{i}(t-d_{E})$  $\Lambda_{i}(t-d_{E})\times(1-\phi_{i})$  $\Lambda_{i}\left( t-d_{E} \right)\times\phi_{i}\times\left( 1-f_{T} \right)$  $\Lambda_{i}(t-d_{E})\times\phi_{i}\times f_{T}$ |
| Progression of untreated disease to asymptomatic infection | D 🡪 A | $r_{D}=\frac{1}{d_{D}}$ |
| Progression of asymptomatic infection to sub-patent infection | A 🡪 U | $r_{A}=\frac{1}{d_{A}}$ |
| Progression of sub-patent infection to susceptible | U 🡪 S | $r_{U}=\frac{1}{d_{U}}$ |
| Progression of treated disease to susceptible (treated individuals experience a period of drug-dependent partial protection from reinfection) | T 🡪 S | $r_{T}=\frac{1}{d_{T}}$ |
| Super-infection from untreated clinical disease, asymptomatic, or sub-patent infection | D 🡪 I  A 🡪 I  U 🡪 I | $\Lambda_{i}(t-d_{E})$ |

**Mosquito biting rates**

Biting rates are dependent on heterogeneity in exposure to bites that occurs at various spatial scales, assigned to an individual at birth, and an age-dependent biting rate, which changes over the life course due to varying risk of exposure via body surface area. Age-dependency is modelled through a changing entomological inoculation rate (EIR), the number of infectious bites per individual per timestep.

The age-dependent biting rate $\psi_{i}$ is calculated as:

$$\psi_{i}\left( a \right)= 1- \rho exp\left( -\frac{a}{a_{0}} \right)$$

Here the parameters $\rho$ and $a_{0}$ determine the relationship between age and biting rate.

An individual’s relative biting rate, $\zeta_{i}$, is drawn from a log-normal distribution with a mean of 1 and is assigned at birth to remain constant over their lifetime:

$$\log\left( \zeta_{i} \right)\sim N\left( \frac{-\sigma^{2}}{2},\sigma^{2} \right)$$

Combining the individual biting rate and the age-specific biting rate gives the entomological inoculation rate (EIR) $\varepsilon_{i}(a,t)$, which is the number of infectious bites per individual per timestep, and the force of infection $\Lambda_{i}(a,t)$ experienced by individual $i$ with age $a$ at time $t$:

$$\varepsilon_{i}\left( a,t \right)= \varepsilon_{0}(t) \zeta_{i}\psi_{i}(a)$$

$$\Lambda_{i}\left( a,t \right)= b_{i}(t) \varepsilon_{i}(a,t)$$

Here $\varepsilon_{0}(t)$ is the mean EIR experienced by adults at time $t$ and $b_{i}(t)$ is the probability that an infectious bite leads to a patent infection, dependent on the level of pre-erythrocytic immunity and given a lag of $d_{E}$ days to account for the latent period of sporozoite infection after an infectious bite.

The mean EIR experienced by adults is calculated as:

| $\varepsilon_{0}(t)= \frac{\alpha I_{M}}{\omega}$ |
| --- |

Where $\alpha$ is the mosquito biting rate on humans, $I_{M}$ is the compartment for adult infectious mosquitoes (see vector model component), and $\omega$ is a normalising constant for the biting rate with age which incorporates a cross-sectional human population age-distribution $g\left( a \right)$:

$$\omega= \int_{0}^{\infty} \psi\left( a \right)g\left( a \right)da$$

**Immunity**

The probability of infection *b*, probability of clinical symptomatic disease *𝜙*, and recovery rate from asymptomatic infection *r_A_*, all depend on immunity levels. The acquisition and loss of naturally-acquired immunity is captured dynamically in the model, driven by both age and exposure. Naturally-acquired immunity affects three different outcomes in the model, leading to: a) a reduced probability of developing a blood-stage infection following an infectious bite due to pre-erythrocytic immunity, $I_{B}$, b) a reduced probability of progression to clinical disease following infection, dependent on exposure-driven and maternally acquired clinical immunity, $I_{CA}$ and $I_{CM}$, and c) a reduced probability of a blood-stage infection being detected by microscopy, dependent on acquired immunity to the detectability of infection, $I_{D}$. The mechanism underlying the different types of immunity are described below.

**Maternal immunity** against clinical disease $I_{CM}$ and severe disease $I_{VM}$ is acquired at birth (placental antibody transfer) and decays exponentially at rates $r_{CM}$ and $r_{VM}$, respectively, to cause a reduction in the probability of clinical or severe disease given an infection, where:

$r_{CM}=\frac{1}{d_{CM}}$, $r_{VM}=\frac{1}{d_{VM}}$

The level of immunity set at birth is a proportion $P_{CM}$ and $P_{VM}$ of the immunity in a representative population aged 15-35 years with the same biting heterogeneity level as the targeted individual.

**Pre-erythrocytic immunity**$I_{B}$ develops in children and is boosted each time an individual receives an infectious bite (if it has been at least $u_{B}$ since the previous exposure) to reduce the probability of infection given an infectious bite. $I_{B}$decays exponentially between exposures at rate $r_{B}$:

$$r_{B}=\frac{1}{d_{B}}$$

**Blood stage immunity** against severe disease $I_{VA}$ and clinical disease $I_{CA}$ reduces parasite densities in the blood and leads to a reduction in the probability of clinical disease and severe disease. Blood stage immunity parameters decay exponentially between exposures at rates:

$r_{VA}=\frac{1}{d_{VA}}$ , $r_{C}=\frac{1}{d_{CA}}$

Each parameter is boosted following each patent infection if it has been at least $u_{V}$ or $u_{C}$ days respectively since the last exposure.

**Detection immunity** (blood stage) against detectability of asymptomatic infection$I_{D}$ reduces both the probability of detection by microscopy and the infectiousness to mosquitoes:

$$r_{ID}=\frac{1}{d_{ID}}$$

Each parameter is boosted following each patent infection if it has been at least $u_{D}$ days respectively since the last exposure.

Hill functions are then used to convert immunity levels to individual time-dependent probabilities of developing an infection. The probability of developing infection upon exposure is represented by the following for individual $i$at time $t$:

$$b_{i}\left( t \right)= b_{0}\left( b_{1}+ \frac{1-b_{1}}{1+\left( \frac{I_{B}(i,t)}{I_{B0}} \right)^{\kappa_{B}}} \right)$$

Where $b_{0}$ is the baseline probability of infection with no immunity, $b_{1}$ is the minimum probability of infection, $I_{B0}$ and $\kappa_{B}$ are shape and scale parameters respectively, and $I_{B}(i,t)$ is the current level of pre-erythrocytic immunity for that specific individual and specific timepoint*.*

The probability of developing clinical disease (conditional on infection) is represented by the following for individual $i$at time $t$:

$$\phi_{i}(t)=\phi_{0}\left( \phi_{1}+\frac{1-\phi_{1}}{1+\left( \frac{I_{CA}(i,t)+I_{CM}(i,t)}{I_{C0}} \right)^{\kappa_{C}}} \right)$$

Where $\phi_{0}$ is the baseline probability of disease with no immunity, $\phi_{1}$ is the minimum probability of developing clinical disease, $I_{C0}$ and $\kappa_{C}$ are scale and shape parameters respectively, $I_{CA}(i,t)$ is the level of acquired immunity to clinical disease, and $I_{CM}(i,t)$ is the level of maternally acquired immunity to clinical disease.

The probability of developing severe disease (conditional on infection) is represented by the following for individual $i$at time $t$:

$$\theta_{i}(a,t)=\theta_{0}\left( \theta_{1}+\frac{1-\theta_{1}}{1+f_{V}\left( i,a \right)\left( \frac{(I_{VA}\left( i,t \right)+I_{VM}\left( i,t \right)}{I_{V0}} \right)^{\kappa_{V}}} \right)$$

Where $\theta_{0}$ is the probability of severe disease with no immunity, $\theta_{1}$ is the minimum probability, $I_{V0}$ and $\kappa_{V}$ are scale and shape parameters respectively, $I_{VA}(i,t)$ is the level of acquired immunity to severe disease, and $I_{VM}(i,t)$ is the level of maternally acquired immunity to severe disease. $f_{V}\left( i,a \right)$ is an age-dependent modifier of the risk of severe disease with $f_{V0},a_{V}$ and $\gamma_{V}$ parameters:

$$f_{V}(i,a)=1-\frac{\left( 1-f_{V0} \right)}{\left( 1+\left( \frac{a}{a_{V}} \right)^{\gamma_{V}} \right)}$$

Treated individuals experience a reduction in the probability of moving from clinical disease to severe disease, $f_{VT}$.

The probability that an asymptomatic infection is detectable by microscopy is represented by:

$$q_{i}(a,t)=d_{1}+\frac{1-d_{min}}{\left( \left( \frac{1+I_{D}\left( i,t \right)}{I_{D0}} \right)^{\kappa_{D}}f_{D}\left( i,a \right) \right)}$$

Where $d_{min}$ is the minimum probability of detection, $I_{D0}$ and $\kappa_{D}$ are scale and shape parameters respectively, $I_{D}(i,t)$ is the level of acquired immunity to the detectability of infection of individual $i$ at time $t$and $f_{D}\left( i,a \right)$ is an age-dependent modifier of the risk of detectability with $f_{D0},a_{D}$ and $\gamma_{D}$ parameters:

$$f_{D}(i,a)=1-\frac{\left( 1-f_{D0} \right)}{\left( 1+\left( \frac{a}{a_{D}} \right)^{\gamma_{D}} \right)}$$

**Severe disease and mortality**

Severe disease and malaria-associated mortality are derived from the model outputs (2, 3). The incidence of severe malaria requiring hospitalisation in the age range $a_{L}$ to $a_{U}$ at time *t* is given by:

$$\lambda_{H}\left( t,\left( a_{L},a_{U} \right) \right)=\frac{\sum_{i:a_{L}<a_{i}\left( t \right)<a_{U}} (\left( 1-f_{T} \right)+f_{T}f_{VT})\Lambda_{i}(t)\theta_{i}(t)}{\#\{i:a_{L}<a_{i}\left( t \right)<a_{U}\}}$$

Where $\Lambda_{i}(t)$ is the force of infection experienced by individual *i* at time *t* and $\theta_{i}\left( t \right)$ is the probability that individual *i* develops severe disease upon being infected. Individuals receiving treatment are assumed to experience a 42.2% reduction in the probability of disease progression to severe disease, $f_{VT}$ (4).

Malaria-related deaths are assumed to be a proportion of those experiencing severe disease:

$$\mu\left( t,\left( a_{L},a_{U} \right) \right)=v\lambda_{H}\left( t,\left( a_{L},a_{U} \right) \right)$$

Where $v$ is a scaling factor, previously estimated at 0.215 (3).

**Human infectivity to mosquitoes**

The reduction in parasite density caused by immunity to detection decreases the probability of transmission of infection to mosquitoes. In states $D$ and $U$, onwards infectiousness is represented by $c_{D}$ and $c_{U}$ respectively, and $c_{T}$ following treatment. In state $A$, infectiousness is modified by $q_{i}$, the detectability of individual $i$: $c_{A}{=c}_{U}+(c_{D}+c_{U}) q_{i}^{\gamma_{1}}$.

### Model parameters

All model parameter values are summarised in **Table S1.2**.

**Table S1.2.** *P. falciparum* model parameter values. Full details can be found in the original publications including references for parameters and intervals for the prior and posterior distributions (median values of the posterior distribution are used in model simulations) (3, 5-9).

| Parameter | Symbol | Estimate |
| --- | --- | --- |
| HUMAN COMPONENT |  |  |
| Human infection duration |  |  |
| Latent period | $d_{E}$ | 12 days |
| Patent infection | $\frac{1}{r_{A}}$ | 195 days |
| Clinical disease (untreated) | $\frac{1}{r_{D}}$ | 5 days |
| Treatment of clinical disease | $\frac{1}{r_{T}}$ | 5 days |
| Sub-patent infection | $\frac{1}{r_{U}}$ | 110 days |
| Age and heterogeneity |  |  |
| Age-dependent biting parameter | $\rho$ | 0.85 |
| Age-dependent biting parameter | $a_{0}$ | 8 years |
| Variance of the log heterogeneity in biting rates | $\sigma^{2}$ | 1.67 |
| Pre-erythrocytic immunity reducing probability of infection |  |  |
| Duration of refractory period in which immunity is not boosted | $u_{B}$ | 7.19919 days |
| Duration of pre-erythrocytic immunity | $d_{B}$ | 10 years |
| Maximum probability of infection due to no immunity | $b_{0}$ | 0.590076 |
| Maximum relative reduction in probability of infection due to immunity | $b_{1}$ | 0.5 |
| Scale parameter | $I_{B0}$ | 43.8787 |
| Shape parameter | $\kappa_{B}$ | 2.15506 |
| Immunity reducing probability of clinical disease |  |  |
| Duration of refractory period in which immunity is not boosted | $u_{C}$ | 6.06349 days |
| Duration of clinical immunity | $d_{CA}$ | 30 years |
| New-born immunity relative to mother’s clinical immunity | $P_{CM}$ | 0.774368 |
| Duration of maternal immunity | $d_{CM}$ | 67.6952 days |
| Maximum probability of clinical disease due to no immunity | $\Phi_{0}$ | 0.791666 |
| Maximum relative reduction in probability of clinical disease due to immunity | $\Phi_{1}$ | 0.000737 |
| Scale parameter | $I_{C0}$ | 18.02366 |
| Shape parameter | $\kappa_{C}$ | 2.36949 |
| Immunity reducing probability of severe disease |  |  |
| Duration of refractory period in which immunity is not boosted | $u_{V}$ | 11.4321 days |
| Duration of severe disease immunity | $d_{VA}$ | 30 years |
| New-born immunity relative to mother’s severe immunity | $P_{VM}$ | 0.195768 |
| Duration of maternal immunity | $d_{VM}$ | 76.8365 days |
| Maximum probability of severe disease due to no immunity | $\theta_{0}$ | 0.0749886 |
| Maximum relative reduction in probability of severe disease due to immunity | $\theta_{1}$ | 0.0001191 |
| Scale parameter | $I_{V0}$ | 1.09629 |
| Shape parameter | $\kappa_{V}$ | 2.00048 |
| Parameter for age-dependent modifier of severe disease risk | $f_{V0}$ | 0.141195 |
| Parameter for age-dependent modifier of severe disease risk | $a_{V}$ | 2493.41 |
| Parameter for age-dependent modifier of severe disease risk | $\gamma_{V}$ | 2.91282 |
| Immunity reducing probability of detection |  |  |
| Duration of refractory period in which immunity is not boosted | $u_{D}$ | 9.44512 days |
| Duration of detection immunity | $d_{ID}$ | 10 years |
| Minimum probability of detection due to maximum immunity | $d_{1}$ | 0.160527 |
| Scale parameter | $I_{D0}$ | 1.577533 |
| Shape parameter | $\kappa_{D}$ | 0.476614 |
| Scale parameter relating age to immunity | $a_{D}$ | 21.9 years |
| Time-scale at which immunity changes with age | $f_{D0}$ | 0.007055 |
| Shape parameter relating age to immunity | $\gamma_{D}$ | 4.8183 |
| VECTOR COMPONENT |  |  |
| Infectiousness of humans to mosquitoes |  |  |
| Lag from parasites to infectious gametocytes | $\tau_{1}$ | 12.5 days |
| Untreated clinical disease | $c_{D}$ | 0.068 |
| Sub-patent infection | $c_{U}$ | 0.0062 |
| Parameter for infectiousness of asymptomatic infection | $\gamma_{1}$ | 1.82425 |
| Mosquito Population Model |  |  |
| Daily mortality of adult mosquitoes with no interventions | $\mu_{0}$ | 0.125 |
| Extrinsic incubation period | $\tau_{M}$ | 10 days |
| Larval model |  |  |
| Early instar larval developmental period | $d_{E}$ | 6.64 days |
| Late instar developmental period | $d_{L}$ | 3.72 days |
| Pupal developmental period | $d_{P}$ | 0.643 days |
| Daily mortality rate of early-stage larvae (density dependent) | $\mu_{E}$ | 0.0338 |
| Daily mortality rate of late-stage larvae (density dependent) | $\mu_{L}$ | 0.0348 |
| Daily mortality rate of pupae (density independent) | $\mu_{P}$ | 0.249 |
| Effect of density dependence on late instars relative to early instars | $\gamma$ | 13.25 |
| Maximum number of eggs per oviposition per mosquito | $\beta_{L_{max}}$ | 21.2 |
| Mosquito behaviour |  |  |
| Mean duration of host-seeking in the absence of vector control interventions | $\delta_{1}$ | 0.69 days |
| Mean duration of resting between blood meals | $\delta_{2}$ | 2.31 days |
| Proportion of bites taken on humans (anthropophagy) in the absence of vector control interventions | $Q_{0}$ | Varies in analysis |
| Proportion of bites taken on humans indoors | $\Phi_{I}$ | Varies in analysis |
| Proportion of bites taken on humans in bed | $\Phi_{b}$ | Varies in analysis |
| Seasonality |  |  |
| Rainfall Fourier parameters: highly seasonal setting | $g_{0}$ | 0.284596 |
|  | $g_{1}$ | -0.317878 |
|  | $g_{2}$ | -0.0017527 |
|  | $g_{3}$ | 0.116455 |
|  | $h_{1}$ | -0.331361 |
|  | $h_{2}$ | 0.293128 |
|  | $h_{3}$ | -0.0617547 |
| Rainfall Fourier parameters: seasonal setting | $g_{0}$ | 0.285505 |
|  | $g_{1}$ | -0.325352 |
|  | $g_{2}$ | -0.0109352 |
|  | $g_{3}$ | 0.0779865 |
|  | $h_{1}$ | -0.132815 |
|  | $h_{2}$ | 0.104675 |
|  | $h_{3}$ | -0.013919 |
| Rainfall Fourier parameters: perennial setting | $g_{0}$ | 0.2852770 |
|  | $g_{1}$ | -0.0248801 |
|  | $g_{2}$ | -0.0529426 |
|  | $g_{3}$ | -0.0168910 |
|  | $h_{1}$ | -0.0216681 |
|  | $h_{2}$ | -0.0242904 |
|  | $h_{3}$ | -0.0073646 |
| Minimum rainfall value | - | 0.001 |
| INTERVENTIONS |  |  |
| Insecticide-treated nets (ITNs) |  |  |
| Maximum probability of a mosquito being repelled by a ITN with full insecticidal and barrier effect | $r_{N0}$ | Varies in analysis, by insecticide and resistance |
| Minimum probability of a mosquito being repelled by a ITN after decay | $r_{NM}$ | 0.24 |
| ITN half-life | $H_{py}$ | Varies in analysis, by insecticide and resistance |
| Maximum probability of a mosquito being killed by a ITN with full insecticidal and barrier effect | $d_{N0}$ | Varies in analysis, by insecticide and resistance |
| Indoor residual spraying (IRS) with Actellic® |  |  |
| Parameter for maximum killing effect on mosquitoes after spraying | $l_{S\vartheta}$ | 2.025 |
| Parameter for waning of killing effect on mosquitoes over time | $l_{S\gamma}$ | -0.009 |
| Parameter for maximum blood-feeding inhibition effect on mosquitoes after spraying | $k_{S\vartheta}$ | -2.222 |
| Parameter for waning of blood-feeding inhibition effect on mosquitoes over time | $k_{S\gamma}$ | 0.008 |
| Parameter for maximum deterrence effect on mosquitoes after spraying | $m_{S\vartheta}$ | -1.232 |
| Parameter for waning of deterrence effect on mosquitoes over time | $m_{S\gamma}$ | -0.009 |
| Treatment with artemether-lumefantrine (AL) |  |  |
| Treatment coverage | $f_{T}$ | Varies in analysis |
| Drug efficacy | - | 0.95 |
| Infectiousness after treatment relative to untreated infection | $c_{T}$ | 0.0509*$c_{D}$ |
| Shape parameter for Weibull curve for drug-dependent prophylaxis | $P_{T}()$ | 11.3 |
| Scale parameter for Weibull curve for drug-dependent prophylaxis |  | 10.6 |
| Seasonal malaria chemoprevention (SMC) with SP+AQ |  |  |
| Drug efficacy | - | 0.90 |
| Infectiousness after treatment relative to untreated infection | $c_{T}$ | 0.3200*$c_{D}$ |
| Shape parameter for Weibull curve for drug-dependent prophylaxis | $k$ | 3.40  With RTS,S synergy: 2.87 |
| Scale parameter for Weibull curve for drug-dependent prophylaxis | $\lambda$ | 39.34  With RTS,S synergy: 45.76 |
| RTS,S vaccine |  |  |
| Peak antibody titer following primary vaccination schedule (mean, standard deviation) | ${CSP}_{peak}$ | 6.37008, 0.35 |
| Peak antibody titer following booster dose (mean, standard deviation) | ${CSP}_{boost}$ | 5.56277, 0.35  With SMC synergy: 6.37008, 0.35 |
| Proportion of short-lived component of antibody response following primary vaccination schedule (mean, standard deviation) | $\rho_{peak}$ | 2.37832, 1.00813 |
| Proportion of short-lived component of antibody response following booster dose (mean, standard deviation) | $\rho_{boost}$ | 1.03431, 1.02735 |
| Half-life of short-lived component of antibody response (mean, standard deviation) | $d_{s}$ | 3.74502, 0.341185 |
| Half-life of long-lived component of antibody response (mean, standard deviation) | $d_{l}$ | 6.30365, 0.396515 |
| Maximum vaccine efficacy | $v_{max}$ | 0.93  With SMC synergy: 0.843 |
| Shape parameter for the vaccine efficacy model | $\alpha$ | 0.74  With SMC synergy: 0.868 |
| Scale parameter for the vaccine efficacy model | $\beta$ | 99.4  With SMC synergy: 70.9 |

### Vector component

The vector model is based on the deterministic compartmental model previously described in White et al. (10) and captures adult mosquito transmission dynamics as well as larval population dynamics. In this analysis, we modelled the characteristics of the average local vector population rather than individual mosquito species.

*Mosquito transmission model*

Adult mosquitoes move between three states, $S_{M}$ (susceptible), $E_{M}$ (exposed), and $I_{M}$ (infectious), as follows:

| $\frac{dS_{M}}{dt}= -\Lambda_{M}S_{M}+ \beta\left( t \right)- \mu S_{M}$  $\frac{dE_{M}}{dt}= \Lambda_{M}S_{M} - \Lambda_{M}\left( t-\tau_{M} \right)S_{M}\left( t-\tau_{M} \right)P_{M}- \mu E_{M}$  $\frac{dI_{M}}{dt}= \Lambda_{M}\left( t-\tau_{M} \right)S_{M}\left( t-\tau_{M} \right)P_{M}- \mu I_{M}$ |
| --- |

$\Lambda_{M}$ is the force of infection from humans to mosquitos, $\beta\left( t \right)$ represents the time-varying adult mosquito emergence rate, $\mu$ is the adult mosquito death rate, and $\tau_{M}$ represents the extrinsic incubation period. $P_{M}$represents the probability that a mosquito survives between being infected and sporozoites appearing in the salivary glands and is calculated as $exp(-\mu\tau_{M})$.

The force of infection experienced by the vector is the sum of the contribution to mosquito infections from all human infectious states. It is represented by:

| $\Lambda_{M}\left( t \right)= \frac{\alpha}{\omega}\iint_{\zeta\alpha} \zeta\psi\left( a \right)\left( c_{D}D\left( \zeta,a,t-\tau_{1} \right)+c_{T}T\left( \zeta,a,t-\tau_{1} \right)+c_{A}A\left( \zeta,a,t-\tau_{1} \right)+ c_{U}U\left( \zeta,a,t-\tau_{1} \right) \right)da d\zeta$ |
| --- |

where $\alpha$ is the biting rate on humans and the parameter $\omega$ represents a normalising constant for the biting rate over all ages, as described for the human model component. $c_{D}$, $c_{T}$, $c_{A}$, and $c_{U}$ represent the human-to-mosquito infectiousness for untreated symptomatic infection, treated symptomatic infection, asymptomatic infection and asymptomatic sub-patent infection, respectively. $\tau_{1}$ is the time-lag between parasitemia with asexual parasite stages and gametocytemia to account for the time to *P. falciparum* gametocyte development.

*Larval development*

Female adult mosquitoes lay eggs at a rate $\beta_{L}$. Upon hatching from eggs, larvae progress through early and late larvae stages ($E$ and $L$ compartments) before developing into to the pupal stage $P_{L}$. Adult female mosquitoes then emerge from the pupal stage, which is calculated as $\beta=0.5 \frac{P_{L}}{d_{P}}$.

| $\frac{dE}{dt}=\beta_{L}\left( S_{M}+E_{M}+I_{M} \right)-\mu_{E}\left( 1+\frac{E+L}{K} \right)E-\frac{E}{d_{E}}$  $\frac{dL}{dt}=\frac{E}{d_{E}}-\mu_{L}\left( 1+\gamma\frac{E+L}{K} \right)L-\frac{L}{d_{L}}$  $\frac{dP_{L}}{dt}=\frac{L}{d_{L}}-\mu_{P}P_{L}-\frac{P_{L}}{d_{P}}$ |
| --- |

The duration of each larval stage is represented by $d_{E}$, $d_{L}$ and $d_{P}$. The larval stages are regulated by density-dependent mortality rates, with a time-varying carrying-capacity, $K(t)$, that represents the ability of the environment to sustain breeding sites through different periods of the year and with the density of larvae in relation to the carrying-capacity regulated by a parameter $\gamma$. The carrying capacity determines the mosquito density and hence the baseline transmission intensity in the absence of interventions. Seasonality is also represented by changes in the environment’s carrying capacity over time. It is calculated as:

$$K\left( t \right)= K_{0}\frac{R(t)}{\bar{R}}$$

Here $K_{0}$ is the baseline carrying capacity, $\bar{R}$ is the mean rainfall over the course of the year and $R(t)$ is the time-varying seasonal curve of interest, obtained from average rainfall data using the first three frequencies of a Fourier transform:

$$R\left( t \right)=g_{0}+\sum_{i=1}^{3} g_{i}\cos(2\pi ti)+h_{i}\sin(2\pi ti)$$

The three seasonality profiles in the simulations were based on data from Central Africa (perennial), West African coastal regions (seasonal) and the Sahel region (highly seasonal) and are illustrated in **Figure S2** (11).


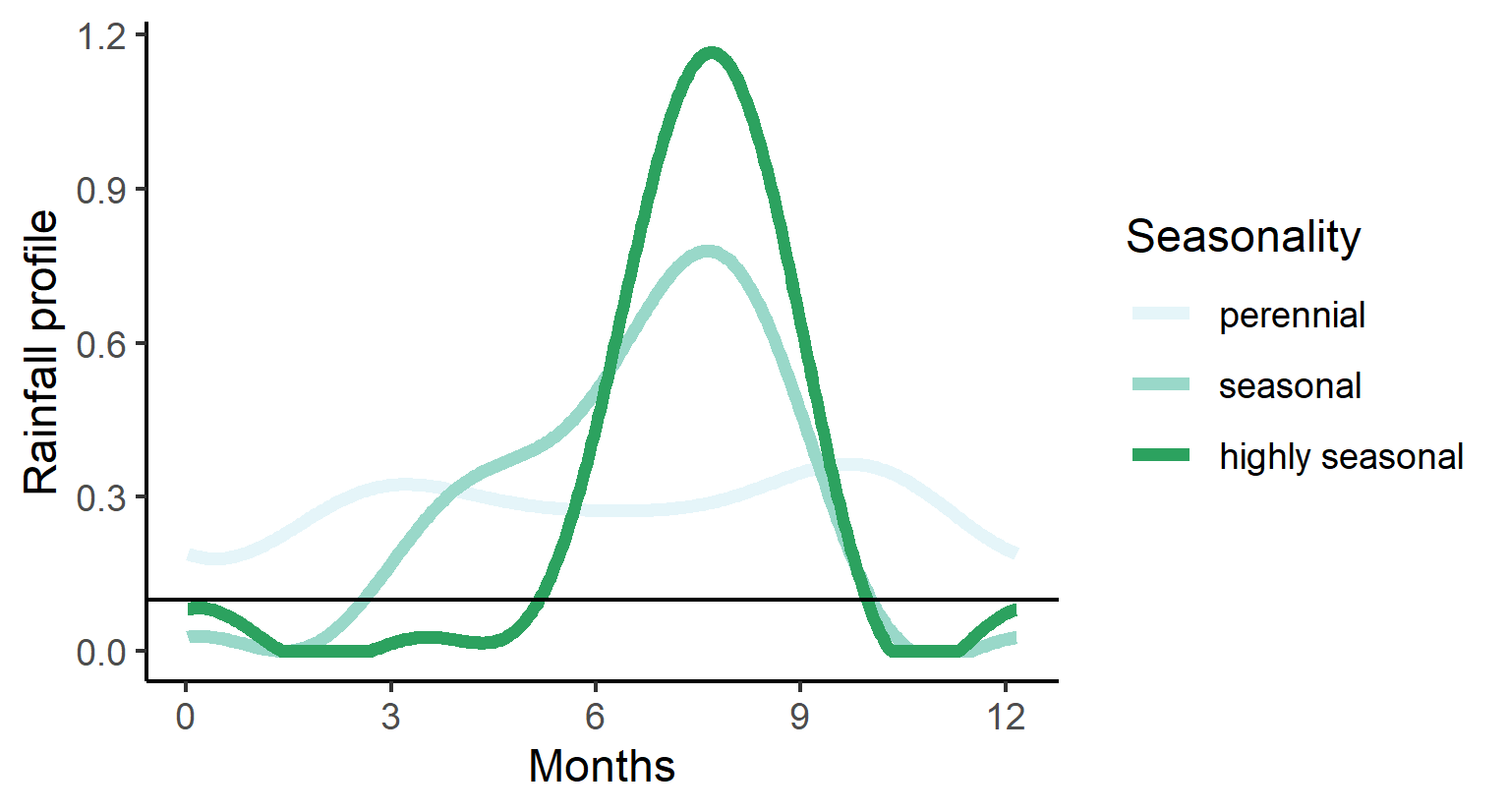


**Figure S1.2. Seasonality profiles in model simulations.**

The baseline carrying capacity is represented by:

| $K_{0}= M_{0}\frac{2d_{L}\mu_{0}(1+d_{P}\mu_{P})\gamma(\lambda_{M}+1)}{(\frac{\lambda_{M}}{\mu_{L}d_{E}}-\frac{1}{\mu_{L}d_{L}}-1)}$ |
| --- |

Where $M_{0}$ is the initial female mosquito density, $\mu_{0}$ is the baseline mosquito death rate and $\lambda_{M}$ is defined as:

| $\lambda_{M}=-0.5\left( \gamma\frac{\mu_{L}}{\mu_{E}}-\frac{d_{E}}{d_{L}}+\left( \gamma-1 \right)\mu_{L}d_{E} \right)+\sqrt{0.25\left( \gamma\frac{\mu_{L}}{\mu_{E}}-\frac{d_{E}}{d_{L}}+\left( \gamma-1 \right)\mu_{L}d_{E} \right)^{2}+\gamma\frac{\beta_{L}\mu_{L}d_{E}}{2\mu_{E}\mu_{0}d_{L}(1+d_{P}\mu_{P})}}$ |  |
| --- | --- |

In this equation, the number of eggs laid per day, $\beta_{L}$, is defined as:

| $\beta_{L}=\frac{\beta_{L_{max}} \mu e^{-\frac{\mu}{f_{R}}}}{\mu(e^{\frac{\mu}{f_{R}}}-1)(1-e^{-\frac{\mu}{f_{R}}})}$ |
| --- |

Where $\beta_{L_{max}}$ is the maximum number of eggs per oviposition per mosquito. The adult mosquito death rate $\mu$ and the mosquito feeding rate $f_{R}$ are affected by the use of ITNs and further described in the following section on modelling vector control. Full details on the derivation of the egg-laying rate $\beta_{L}$ and the carrying capacity $K$ have been previously published (10).

### Interventions

The model supports a range of anti-malaria interventions included in this analysis, namely treatment, insecticide-treated nets (ITNs), indoor residual spraying (IRS), seasonal malaria chemoprevention (SMC) and the RTS,S vaccine.

**Treatment**

A treated individual moves from a clinically diseased state to a susceptible state and retains a drug-dependent period of partial protection from infection. The first-line drug artemether-lumefantrine (AL) used here has a 95% probability of successfully clearing infection and protection was originally characterised using a pharmacokinetic-pharmacodynamic model fit to clinical trial data from six locations across sub-Saharan Africa (6).

The protection from infection at time *u* after effective treatment is represented by $P_{T}(u)$ and the probability of re-infection is multiplied by $1-P_{T}(u)$. The overall level of protection is found by taking the area under the curve:

$$\begin{aligned} A_{T}=\int_{0}^{\infty} P_{T}\left( u \right) du \end{aligned}$$

Treatment with AL results in an overall level of protection, $A_{T},$ varying from 7 to 16 days (depending on age).

**Vector control**

Insecticide-treated bed nets (ITNs) and indoor residual spraying (IRS) are modelled probabilistically as malaria prevention tools by either repelling female mosquitoes from biting a human host or by killing mosquitoes (3, 7).

*Mosquito feeding behavior*

There are six possible outcomes of an attempted mosquito feed:

1. The mosquito bites a non-human host.
2. The mosquito is killed by the ITN before biting.
3. The mosquito is killed by IRS before biting.
4. The mosquito is killed by IRS after it bites.
5. The mosquito successfully feeds and survives.
6. The mosquito is repelled without biting (either by ITNs or IRS) and will attempt to find an alternative blood meal source.

The probability that a blood-seeking mosquito successfully feeds on a human depends on species-dependent bionomics and behaviors of the mosquito, as well as the anti-vectoral interventions present in the human population. Parameter values can be found in **Table S1.2**.

We define the probability of a mosquito biting a human host during a single attempt as $y_{i}$, the probability that a mosquito bites a human host and survives the feeding attempt as $w_{i}$, and the probability of a mosquito being repelled without feeding as $z_{i}$. These probabilities exclude natural vector mortality, so that for a population without protection from ITNs (e.g. prior to their introduction), $y_{1}=w_{1}=1$ and $z_{1}=0$.

The presence of ITNs and/or IRS modifies these probabilities of surviving a feeding attempt or being repelled without feeding. Upon entering a house with ITNs, mosquitoes can experience three different outcomes: being repelled by the ITN without feeding (probability $r_{N}$), being killed by the ITN before biting (probability $d_{N}$), or feeding successfully (probability $s_{N}$). It is assumed that all biting attempts indoors occur on humans. The repellency of ITNs in terms of the insecticide and barrier effect decays over time, giving the following probabilities:

| $r_{N}=\left( r_{N0}-r_{NM} \right)\exp\left( -t\gamma_{N} \right)+r_{NM}$ |  |
| --- | --- |
| $d_{N}=d_{N0}\exp\left( -t\gamma_{N} \right)$ |  |
| $s_{N}=1-r_{N}-d_{N}$ |  |

Where $r_{N0}$ is the maximum probability of a mosquito being repelled by a bednet and $r_{NM}$ is the minimum probability of being repelled by a bednet that no longer has insecticidal activity and possibly holes reducing the barrier effect. $\gamma_{N}$ represents the rate of decay of the effect of ITNs over time $t$ since their distribution and is derived from the ITN half-life as $\frac{log(2)}{H_{py}}$. The killing effect of ITNs decreases at the same constant rate from a maximum probability of $d_{N0}$. In model simulations, ITNs are distributed every three years.

Mosquitoes can experience the same three outcomes upon an encounter with IRS (7): being repelled without feeding (probability $r_{S}$), being killed before biting (probability $d_{S}$), or feeding successfully (probability $s_{S}$). First, the change in IRS impact over time since spraying was modelled by fitting flexible logistic functions to experimental hut trial data to estimate the proportion of mosquitoes dying after entering a hut, $l_{s}$, the proportion of mosquitoes successfully feeding after entering a hut, $k_{s}$, and the proportion of mosquitoes being repelled after entering a hut, $j_{S}$, according to the following model:

$$l_{s}=\frac{1}{1+e^{-(l_{s\vartheta}+l_{s\gamma}t)}}$$

$$k_{s}=\frac{1}{1+e^{-(k_{s\vartheta}+k_{s\gamma}t)}}$$

$$j_{s}=1-l_{s}-k_{s}$$

Where parameters with subscript $\vartheta$ denote initial efficacy and those with subscript $\gamma$ denote changes in efficacy over time.

Similarly, the proportion of mosquitoes being deterred away from a sprayed hut was estimated by:

$$m_{s}=\frac{1}{1+e^{-(m_{s\vartheta}+m_{s\gamma}t)}}$$

Functions $k_{S}$, $l_{S}$ and $j_{S}$ were adjusted by this deterrence parameter, $m_{S}$, since the probabilities of being killed or successfully feeding are conditional on not being deterred before entering a sprayed hut:

$${k^{'}}_{S}=k_{s}\left( 1-m_{s} \right)$$

$${l^{'}}_{S}=l_{s}\left( 1-m_{s} \right)$$

$${j^{'}}_{S}=j_{s}\left( 1-m_{s} \right)+m_{s}$$

This gives the following probabilities of being repelled without feeding, being killed before biting, and feeding successfully after encountering IRS:

| $r_{S}=\left( 1-\frac{{k^{'}}_{S}}{k_{0}} \right)\left( \frac{{j^{'}}_{S}}{{j^{'}}_{S}+{l^{'}}_{S}} \right)$ |  |
| --- | --- |
| $d_{S}=\left( 1-\frac{{k^{'}}_{S}}{k_{0}} \right)\left( \frac{{l^{'}}_{S}}{{l^{'}}_{S}+{j^{'}}_{S}} \right)$ |  |
| $s_{S}=\frac{{k^{'}}_{S}}{k_{0}}$ |  |

This model allows for the option of a prolonged duration of maximum efficacy of new long-lasting IRS products, and changes in endophily (the propensity for a mosquito to rest indoors following bloodfeeding) based on spraying (7). Median parameter estimates for the entomological efficacy of long-lasting IRS (Actellic®300CS product) were previously obtained by fitting to experimental hut trial data (7). Actellic 300CS was used in simulations because it is the most used IRS insecticide in PMI VectorLink country programmes (12). The calculations for the probability of successful feeding, biting and repellency depend on the combination of ITNs/IRS in place in the household where the individual resides and are shown in **Table S1.3**. Here, $\Phi_{I}$ and $\Phi_{B}$ are the proportions of bites taken on humans whilst indoors and whilst in bed, respectively, which were varied in simulations.

**Table S1.3. Probabilities of successful feeding, biting and repulsion for combinations of ITN/IRS interventions.**

|  | ITN only | IRS only | ITN and IRS |
| --- | --- | --- | --- |
| Probability of successful feeding (*w_i_*) | $1-\Phi_{B}+\Phi_{B}s_{N}$ | $1-\Phi_{I}+\Phi_{I}(1-r_{S})s{}_{S}$ | $\begin{matrix} 1-\Phi_{I}+\Phi_{ B}(1-r_{S})s_{N}s_{S} \\ +(\Phi_{I}-\Phi_{B})(1-r_{S})s_{S} \end{matrix}$ |
| Probability of biting (*y_i_*) | $1-\Phi_{B}+\Phi_{B}s_{N}$ | $1-\Phi_{I}+\Phi_{I}(1-r_{S})$ | $\begin{matrix} 1-\Phi_{I}+\Phi_{B}(1-r_{S})s_{N} \\ +(\Phi_{I}-\Phi_{B})(1-r_{S}) \end{matrix}$ |
| Probability of repellency (*z_i_*) | $\Phi_{B}r{}_{N}$ | $\Phi_{I}r_{S}$ | $\Phi_{B}(1-r_{S})r_{N}+\Phi_{I}r_{S}$ |

During a single feeding attempt (which may be on animals or humans), the probability of a mosquito feeding or being repelled without feeding, $W$ and $Z$, are then:

| $W =1-Q_{0}+Q_{0}\sum_{i} \pi_{i}w_{i}$ |  |
| --- | --- |
| $Z=Q_{0}\sum_{i} \pi_{i}z_{i}$ |  |

Where $Q_{0}$ is the proportion of bites taken on humans in the absence of any vector control intervention and $\pi_{i}$ is the proportion of bites on humans that person *i* receives in the absence of interventions.

*Effect of vector control on mosquito mortality*

The probability of mosquitoes being repelled without feeding in the model affect the mosquito feeding rate, $f_{R}$, as follows:

| $f_{R}=\frac{1}{\frac{\delta_{1}}{(1-Z)}+\delta_{2}}$ |  |
| --- | --- |

Where $\delta_{1}$ is the time spent looking for a blood meal in the absence of vector control, and $\delta_{2}$ is the time spent resting between blood meals, which is assumed to be unaffected by ITN usage.

The average probabilities of feeding or being repelled also affect the probability of surviving the period of feeding, $p_{1}$, as follows:

| $p_{1}=\frac{Wexp(-\mu_{0}\delta_{1})}{1-Zexp(-\mu_{0}\delta_{1})}$ |  |
| --- | --- |

Where $\mu_{0}$ is the baseline mosquito death rate in the absence of interventions.

The probability of surviving the period of resting, $p_{2}$, is not affected by ITNs:

| $p_{2}=exp(-\mu_{0}\delta_{2})$ |  |
| --- | --- |

This allows to calculate the mosquito mortality rate affecting mosquito population dynamics:

| $\mu=-f_{R}ln(p_{1}*p_{2})$ |  |
| --- | --- |

*Effect of vector control on the force of infection acting on humans and mosquitoes*

In the presence of ITNs, the anthropophagy (the proportion of successful bites which are on humans) of mosquitoes is represented by parameter $Q$. This is affected by ITN usage as follows:

| $Q=1-\frac{1-Q_{0}}{W}$ |  |
| --- | --- |

| This then gives the biting rate on humans, $\alpha$:  $\alpha=Qf_{R}\frac{\pi_{i}w_{i}}{\sum_{i} \pi_{i}w_{i}}$ |  |
| --- | --- |

The biting rate at which person *i* is bitten by a mosquito is:

$$\lambda_{i}=\alpha\frac{\pi_{i}w_{i}}{\sum_{i} \pi_{i}w_{i}}$$

In the presence of indoor residual spraying, some mosquitoes bite before being killed by the insecticide while resting on the walls of the house. Therefore, the effective biting rate on each person needs to be inflated by a factor $\frac{y_{i}}{w_{i}}$ , giving:

$$\tilde{\lambda_{i}}=\alpha\frac{\pi_{i}y_{i}}{\sum_{i} \pi_{i}w_{i}}$$

The EIR experienced by person *i* is then $\tilde{\lambda_{i}}I_{M}$.

*Effect of vector control on larval development*

The mosquito death rate $\mu$ and the feeding rate $f_{R}$ also influence the calculation of the carrying capacity $K$ and the egg-laying rate $\beta_{L}$, thereby affecting larval development.

**Seasonal malaria chemoprevention**

SMC is implemented in the model using sulfadoxine-pyrimethamine and amodiaquine (SP+AQ) targeted at children between 3-59 months of age. Doses are timed to overlap the months of highest transmission intensity. Like in the treatment model, SP+AQ directly treats existing infections with a given probability, leading to clearance of infection and return to the susceptible state among infected individuals. Additionally, it provides a period of drug-dependent prophylaxis to both infected and uninfected individuals. The probability that a treated individual is protected from clinical malaria at time $t$ is defined by $P_{SPAQ}\left( t \right)$ using a Weibull cumulative distribution function (9, 13):

$$P_{SPAQ}\left( t \right)= \exp^{-\left( t/\lambda\right)^{k}}$$

where $k$ and $\lambda$ are the shape and scale parameters.

Synergy between SMC and RTS,S was also incorporated based on trial data from Burkina Faso and Mali, with both interventions in combination performing better than either intervention alone. Estimates for synergistic SMC and RTS,S parameters were drawn from fits performed using the model described here alongside the trial data (9, 14).

**Vaccination**

The RTS,S vaccine model was previously fitted to immunogenicity and efficacy data from the Phase III trial set across 11 sites in sub-Saharan Africa (8, 15). Anti-cirumsporozoite protein (CSP) antibodies following vaccination are assumed to follow a biphasic exponential decay function. Following three primary doses of RTS,S, antibody titers peak $\left( {CSP}_{peak} \right)$ and then decline over time $t$with short-lived ($r_{s}$) and long-lived ($r_{l}$) decay rates:

$$CSP\left( t \right)= {CSP}_{peak}\left( \rho_{peak}e^{-r_{s}t}+\left( 1- \rho_{peak} \right)e^{-r_{l}t} \right) for t<t_{boost}$$

$$r_{s}={log}_{e}\left( 2 \right)/d_{s}$$

$$r_{l}={log}_{e}\left( 2 \right)/d_{l}$$

where $d_{s}$ and $d_{l}$ are the half-lives of the short and long-lived cells respectively. The proportion of antibodies generated by short-lived and long-lived cells are represented by $\rho_{peak}$ and $1- \rho_{peak}$ respectively.

A distinct set of parameters for the peak titer (${CSP}_{boost})$and proportion of short-lived antibodies ($\rho_{boost}$) describes the boosting and waning of antibody titres following the fourth dose given at time $t_{boost}$, according to the following equation:

$$CSP\left( t \right)={CSP}_{boost}\left( \rho_{boost}e^{-r_{s}\left( t-t_{boost} \right)}+\left( 1- \rho_{boost} \right)e^{-r_{l}\left( t-t_{boost} \right)} \right) for t\geq t_{boost}$$

Antibody dynamics over time were then used to estimate vaccine efficacy against infection using the following dose-response curve:

$$V\left( t \right)= V_{max} \left( 1-\frac{1}{1+ \left( \frac{(CSP(t)}{\beta} \right)^{\alpha}} \right)$$

where $V_{max}$ represents the maximum efficacy, and $\alpha$ and $\beta$ are shape and scale parameters, respectively.

As mentioned above, synergy between SMC and RTS,S was also incorporated based on trial data, with both interventions in combination performing better than either intervention alone.

## Baseline settings and intervention schedules

The median sub-national parasite prevalence in endemic sub-Saharan African countries in 2022 was estimated at 12% (IQR 4-25, range 0-60%) (16). A 40% prevalence corresponds to the 95^th^ percentile. Bands of 5%, 10%, 20% and 40% were chosen to be broadly representative of current malaria epidemiology in sub-Saharan Africa, and low-transmission/near-elimination settings were excluded due to differing control strategies. To represent baseline settings with a given parasite prevalence in 2-10 year olds, the model was calibrated to target an average prevalence of 5%, 10%, 20% or 40% at equilibrium over a 3 year period in this age cohort, accounting for existing interventions.

Baseline ITN use bands of 20%, 40% and 60% approximately correspond to the distribution of sub-national ITN use estimates in sub-Saharan Africa in 2022 (median 48%, IQR 25-66%) (17). ITNs were assumed to be distributed at random to a proportion of the general population every 3 years, according to standard distribution schedules. When ITNs are distributed, they provide immediate protection to users and are assumed to be adopted directly by the recipients. Each timestep on from distribution, two things happen. The mortality (and repellence) inducing efficacy wanes at the rate defined above (Vector control section), and the adherence to using the ITNs across the population wanes. As adherence to ITNs wanes over time, the modelled ITN usage that is entered as a parameter corresponds to the proportion of the population using nets each night immediately following the first mass campaign. People in the simulated population who do not receive a new net may still use an old ITN from a previous campaign. Therefore, we assumed that only one ITN type would be newly distributed in the analysis, but some pyrethroid-only ITNs remain in use in the population after switching to new nets until they decay as per the efficacy and adherence rates noted.

For this generalised analysis, we focused on simulating core intervention packages. We included all interventions with a strong recommendation in the WHO guidelines for malaria, except those specific to the special risk group of pregnant women (18). Conditionally recommended interventions, such as larviciding, were not modelled as their evidence base is more limited or because they are only applicable in specific locations.

For IRS, which involves spraying of residual insecticides on indoor surfaces, we assumed this would employ non-pyrethroid IRS products which are already most widely used (7). It was applied so that 80% of the population are protected at random each year, but we assumed a correlation in the use of bed nets and IRS in scenarios where these are co-deployed.

SMC involves monthly administration of sulphadoxine-pyrimethamine plus amodiaquine (SP + AQ) to children aged 3-59 months per current World Health Organization recommendations (18). We allowed for administration of 4 and 5 doses in highly seasonal and seasonal settings, respectively, centered around peak seasonality, with 85% coverage (19).

Age-based RTS,S was administered at 85% coverage and according to Malaria Vaccine Implementation Programme guidelines, with three doses at 6, 7.5, and 9 months of age, and an additional dose at 24 months (19, 20).

## Parameter sets for entomological efficacy of ITNs

The 10 posterior parameter sets used for the entomological efficacy parameters for each resistance level and net class in model simulations are shown in **Table S1.4**. The corresponding estimated mosquito mortality in experimental hut trials is shown in **Figure S1.3**. Note that the decay rates for the different ITNs (as represented by the half-life, *H_py_*) were highly uncertain due to the lack of data on how new nets age under field conditions (21). As data on the durability of pyrethroid-pyrrole nets was not yet available, these draws were assumed to arise from the same distribution as for pyrethroid-only nets (22).

**Table S1.4. Parameters for entomological efficacy of ITNs.**

|  |  | Pyrethroid-only nets | | | Pyrethroid-PBO nets | | | Pyrethroid-pyrrole nets | | |
| --- | --- | --- | --- | --- | --- | --- | --- | --- | --- | --- |
| Insecticide resistance (proportion) | Sample number | *d_N0_* | *r_N0_* | *H_py_* | *d_N0_* | *r_N0_* | *H_py_* | *d_N0_* | *r_N0_* | *H_py_* |
| 0 | 1 | 0.330 | 0.646 | 2.640 | 0.485 | 0.510 | 2.640 | 0.534 | 0.464 | 2.640 |
| 0 | 2 | 0.408 | 0.581 | 2.640 | 0.537 | 0.461 | 2.640 | 0.560 | 0.439 | 2.640 |
| 0 | 3 | 0.324 | 0.651 | 2.640 | 0.484 | 0.511 | 2.640 | 0.527 | 0.470 | 2.640 |
| 0 | 4 | 0.325 | 0.650 | 2.640 | 0.480 | 0.515 | 2.640 | 0.538 | 0.460 | 2.640 |
| 0 | 5 | 0.291 | 0.675 | 2.640 | 0.446 | 0.546 | 2.640 | 0.516 | 0.481 | 2.640 |
| 0 | 6 | 0.416 | 0.573 | 2.640 | 0.550 | 0.449 | 2.640 | 0.568 | 0.431 | 2.640 |
| 0 | 7 | 0.341 | 0.637 | 2.640 | 0.491 | 0.505 | 2.640 | 0.547 | 0.452 | 2.640 |
| 0 | 8 | 0.411 | 0.578 | 2.640 | 0.544 | 0.455 | 2.640 | 0.564 | 0.435 | 2.640 |
| 0 | 9 | 0.295 | 0.673 | 2.640 | 0.452 | 0.540 | 2.640 | 0.513 | 0.484 | 2.640 |
| 0 | 10 | 0.315 | 0.658 | 2.640 | 0.473 | 0.521 | 2.640 | 0.534 | 0.464 | 2.640 |
| 0.2 | 1 | 0.309 | 0.662 | 2.505 | 0.465 | 0.528 | 2.404 | 0.523 | 0.474 | 2.483 |
| 0.2 | 2 | 0.384 | 0.601 | 2.431 | 0.524 | 0.474 | 2.461 | 0.553 | 0.446 | 2.555 |
| 0.2 | 3 | 0.310 | 0.661 | 2.551 | 0.470 | 0.524 | 2.479 | 0.520 | 0.477 | 2.502 |
| 0.2 | 4 | 0.310 | 0.661 | 2.542 | 0.465 | 0.528 | 2.464 | 0.531 | 0.467 | 2.537 |
| 0.2 | 5 | 0.285 | 0.680 | 2.603 | 0.438 | 0.553 | 2.559 | 0.511 | 0.485 | 2.443 |
| 0.2 | 6 | 0.399 | 0.588 | 2.471 | 0.541 | 0.457 | 2.523 | 0.564 | 0.435 | 2.597 |
| 0.2 | 7 | 0.316 | 0.657 | 2.469 | 0.467 | 0.527 | 2.356 | 0.535 | 0.463 | 2.516 |
| 0.2 | 8 | 0.385 | 0.600 | 2.409 | 0.530 | 0.468 | 2.454 | 0.557 | 0.442 | 2.565 |
| 0.2 | 9 | 0.280 | 0.683 | 2.555 | 0.435 | 0.556 | 2.450 | 0.504 | 0.493 | 2.502 |
| 0.2 | 10 | 0.309 | 0.662 | 2.600 | 0.466 | 0.527 | 2.564 | 0.531 | 0.467 | 2.564 |
| 0.4 | 1 | 0.282 | 0.682 | 2.358 | 0.436 | 0.556 | 2.113 | 0.506 | 0.490 | 2.315 |
| 0.4 | 2 | 0.353 | 0.628 | 2.197 | 0.501 | 0.495 | 2.192 | 0.542 | 0.457 | 2.451 |
| 0.4 | 3 | 0.292 | 0.674 | 2.449 | 0.451 | 0.541 | 2.276 | 0.509 | 0.487 | 2.353 |
| 0.4 | 4 | 0.290 | 0.676 | 2.430 | 0.444 | 0.548 | 2.244 | 0.520 | 0.478 | 2.423 |
| 0.4 | 5 | 0.276 | 0.686 | 2.557 | 0.429 | 0.562 | 2.457 | 0.506 | 0.491 | 2.221 |
| 0.4 | 6 | 0.374 | 0.609 | 2.274 | 0.528 | 0.470 | 2.342 | 0.558 | 0.441 | 2.543 |
| 0.4 | 7 | 0.283 | 0.681 | 2.289 | 0.430 | 0.561 | 2.007 | 0.517 | 0.480 | 2.374 |
| 0.4 | 8 | 0.350 | 0.629 | 2.155 | 0.506 | 0.490 | 2.163 | 0.545 | 0.453 | 2.477 |
| 0.4 | 9 | 0.260 | 0.697 | 2.460 | 0.410 | 0.578 | 2.224 | 0.490 | 0.506 | 2.336 |
| 0.4 | 10 | 0.300 | 0.668 | 2.550 | 0.458 | 0.535 | 2.466 | 0.526 | 0.471 | 2.477 |
| 0.6 | 1 | 0.244 | 0.707 | 2.195 | 0.388 | 0.598 | 1.763 | 0.477 | 0.517 | 2.136 |
| 0.6 | 2 | 0.305 | 0.665 | 1.938 | 0.458 | 0.535 | 1.783 | 0.518 | 0.479 | 2.319 |
| 0.6 | 3 | 0.267 | 0.692 | 2.325 | 0.421 | 0.568 | 2.010 | 0.493 | 0.503 | 2.191 |
| 0.6 | 4 | 0.263 | 0.695 | 2.297 | 0.411 | 0.578 | 1.962 | 0.501 | 0.496 | 2.295 |
| 0.6 | 5 | 0.264 | 0.694 | 2.498 | 0.414 | 0.575 | 2.321 | 0.497 | 0.499 | 1.971 |
| 0.6 | 6 | 0.338 | 0.639 | 2.039 | 0.501 | 0.495 | 2.041 | 0.545 | 0.454 | 2.474 |
| 0.6 | 7 | 0.237 | 0.712 | 2.100 | 0.368 | 0.614 | 1.609 | 0.482 | 0.513 | 2.205 |
| 0.6 | 8 | 0.298 | 0.671 | 1.881 | 0.457 | 0.536 | 1.708 | 0.518 | 0.479 | 2.366 |
| 0.6 | 9 | 0.234 | 0.714 | 2.347 | 0.373 | 0.610 | 1.954 | 0.468 | 0.526 | 2.128 |
| 0.6 | 10 | 0.289 | 0.677 | 2.485 | 0.445 | 0.547 | 2.333 | 0.519 | 0.478 | 2.370 |
| 0.8 | 1 | 0.184 | 0.740 | 2.011 | 0.301 | 0.668 | 1.376 | 0.417 | 0.572 | 1.946 |
| 0.8 | 2 | 0.224 | 0.719 | 1.658 | 0.355 | 0.625 | 1.220 | 0.457 | 0.536 | 2.126 |
| 0.8 | 3 | 0.226 | 0.719 | 2.163 | 0.364 | 0.618 | 1.644 | 0.459 | 0.535 | 2.011 |
| 0.8 | 4 | 0.217 | 0.724 | 2.127 | 0.348 | 0.631 | 1.592 | 0.461 | 0.532 | 2.143 |
| 0.8 | 5 | 0.244 | 0.707 | 2.409 | 0.388 | 0.598 | 2.112 | 0.482 | 0.513 | 1.691 |
| 0.8 | 6 | 0.273 | 0.688 | 1.749 | 0.433 | 0.558 | 1.493 | 0.510 | 0.487 | 2.368 |
| 0.8 | 7 | 0.165 | 0.749 | 1.906 | 0.262 | 0.695 | 1.235 | 0.407 | 0.581 | 1.992 |
| 0.8 | 8 | 0.209 | 0.728 | 1.601 | 0.338 | 0.640 | 1.116 | 0.445 | 0.547 | 2.210 |
| 0.8 | 9 | 0.190 | 0.738 | 2.206 | 0.309 | 0.662 | 1.630 | 0.426 | 0.564 | 1.845 |
| 0.8 | 10 | 0.269 | 0.691 | 2.387 | 0.422 | 0.568 | 2.118 | 0.506 | 0.490 | 2.227 |
| 1 | 1 | 0.000 | 0.746 | 1.753 | 0.085 | 0.767 | 1.028 | 0.183 | 0.741 | 1.724 |
| 1 | 2 | 0.000 | 0.746 | 1.369 | 0.085 | 0.766 | 0.789 | 0.191 | 0.737 | 1.491 |
| 1 | 3 | 0.000 | 0.746 | 1.781 | 0.082 | 0.767 | 1.033 | 0.198 | 0.733 | 1.773 |
| 1 | 4 | 0.000 | 0.746 | 1.774 | 0.084 | 0.767 | 1.052 | 0.184 | 0.740 | 1.889 |
| 1 | 5 | 0.000 | 0.746 | 1.924 | 0.089 | 0.766 | 1.232 | 0.191 | 0.737 | 1.375 |
| 1 | 6 | 0.000 | 0.746 | 1.324 | 0.077 | 0.767 | 0.730 | 0.186 | 0.739 | 1.795 |
| 1 | 7 | 0.000 | 0.746 | 1.699 | 0.079 | 0.767 | 0.996 | 0.187 | 0.739 | 1.597 |
| 1 | 8 | 0.000 | 0.746 | 1.352 | 0.081 | 0.767 | 0.757 | 0.180 | 0.742 | 1.732 |
| 1 | 9 | 0.000 | 0.746 | 1.909 | 0.081 | 0.767 | 1.192 | 0.197 | 0.734 | 1.279 |
| 1 | 10 | 0.000 | 0.746 | 1.821 | 0.084 | 0.767 | 1.089 | 0.189 | 0.738 | 1.854 |


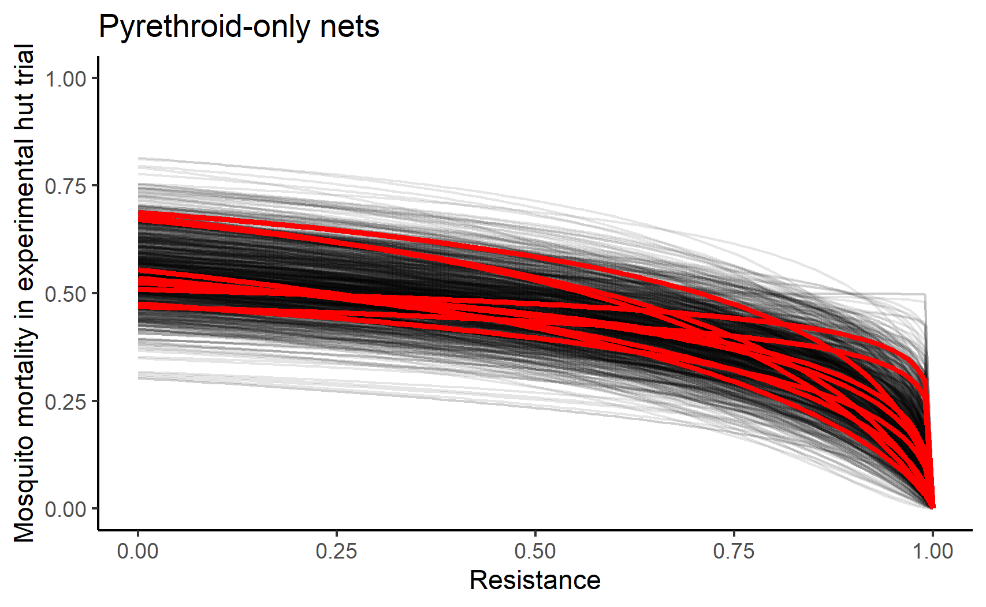

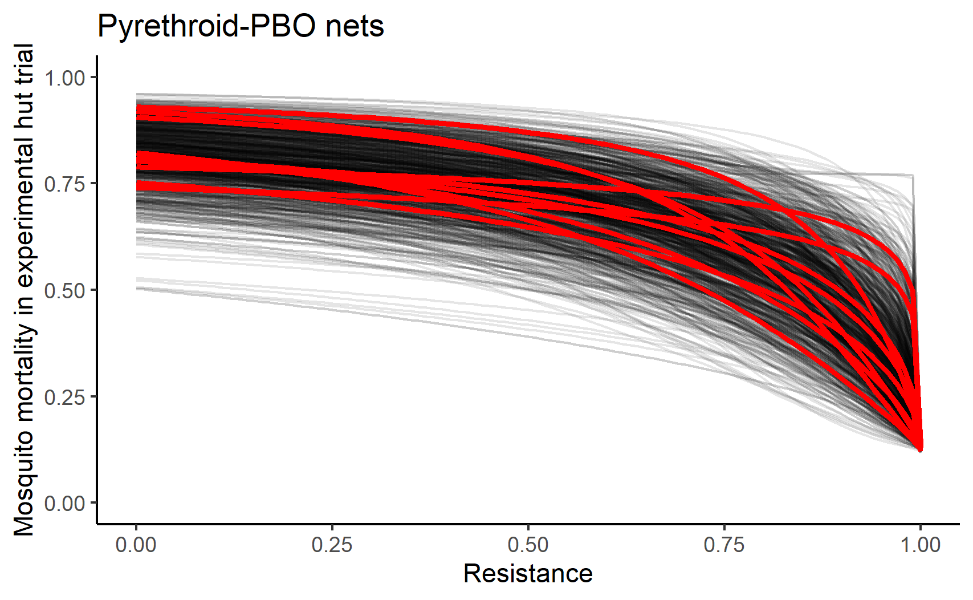

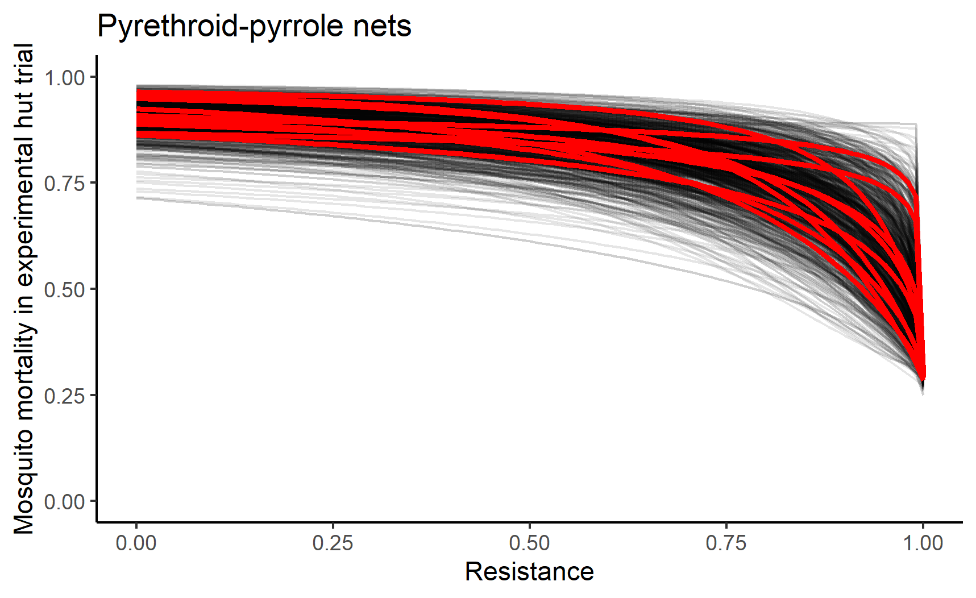


**Figure S1.3. Fitted experimental hut trial mosquito mortality across insecticide resistance levels.** Red lines show outputs from the 10 parameter sets in the analysis and black lines show outputs from 1000 draws from the posterior.

## Calculation of DALYs

DALYs represent the years of life lost due to malaria-related mortality and the morbidity associated with malaria cases. Years of life lost were calculated by multiplying the number of malaria deaths at a given age by an assumed fixed life expectancy of 64.5 years (the 2019 average life expectancy at birth in Africa) (23). Years lived with disability were calculated by assuming average durations of clinical and severe episodes of 5 and 17.5 days, respectively, and using disability weights from the Global Burden of Disease Study 2017 (24). Disability weights were 0.006 for mild malaria (assumed to apply to all clinical cases in those aged over 5 years), 0.051 for moderate severe malaria (assumed to apply to all clinical cases in those aged under 5 years) and 0.133 for severe malaria (assumed to apply to all severe cases).

## Costing and cost-effectiveness thresholds

Intervention costs were estimated in 2023 US dollars using secondary data sources and are detailed in **Table S1.5.** We took a healthcare provider perspective, including donor funding for costs that would otherwise fall on the health budget (25). Costing data from earlier years were converted to 2023 USD using the GDP implicit price deflator from the International Monetary Fund World Economic Outlook (26). No discounting was applied to costs or health outcomes over the short 3-year time horizon. Further details on costing of treatment and vaccines can be found in previous publications (1, 27).

We derived a representative range for cost-effectiveness thresholds based on estimated health opportunity costs (28). Ochalek *et al* generated country-specific estimates of cost per DALY averted ‘thresholds’ in 2015 US$ that reflect health opportunity costs, and also expressed these as a percent of the GDP per capita. Applying these country-specific percentages to the GDP per capita in 2023, the median estimated threshold across malaria-endemic sub-Saharan African countries was US$393 (IQR 181-687, range 69-6,735). Our thresholds of US$250 and US$500 approximately correspond to the 33^rd^ and 66^th^ percentile.

**Table S1.5. Unit costs for malaria interventions.**

| **Intervention** | **Item** | **Unit cost (2023 US$)** | **Source** | **Details** | **Total intervention cost (2023 US$)** |
| --- | --- | --- | --- | --- | --- |
| **Insecticide-treated nets** | Net | Pyrethroid-only: 2.12  Pyrethroid-PBO: 3.01  Pyrethroid-pyrrole: 3.44 | Global Fund Pooled Procurement Mechanism Reference Pricing (Q2 2023) (29) | Median of all Standard Reference prices for pyrethroid-only and pyrethroid-PBO. The price of pyrethroid-pyrrole nets was not known yet; the estimate was derived from the highest PBO net price. | Pyrethroid-only: 3.62 per net distributed  Pyrethroid-PBO:  4.51 per net distributed  Pyrethroid-pyrrole:  4.94 per net distributed |
|  | Delivery | 1.50 | Assumption |  |  |
| **Treatment:**  **clinical cases** | Rapid diagnostic test per person | 0.55 | Global Fund Pooled Procurement Mechanism Reference Pricing (Q2 2023) (30),  Patouillard et al. 2017 (31) | Mean cost from “Reference price per test EXW, USD” column = $0.48. Includes 15% delivery mark-up based on Patouillard et al. 2017. | 10.36 per adult case  6.20 per child case  after removing 23% of treatment costs assumed to represent out-of-pocket payments (32) |
|  | Treatment with Artemether/Lumefantrine per dose | 0.45 | Global Fund Pooled Procurement Mechanism Reference Pricing: Antimalarial medicines (Q2 2023) (33),  WHO guidelines for malaria 2022 (34) | Mean cost for Artemether/Lumefantrine 20/120mg, “Reference price US$ per treatment”  24 doses for adults and 12 doses for children |  |
|  | Outpatient treatment per person | 2.1 | WHO CHOICE (35) | Median “Cost per outpatient visit, Results in 2010 USD, Model prediction” for all malaria-endemic countries in sub-Saharan Africa and across all types of health centers = $1.55. Converted to 2023 US$. |  |
| **Treatment:**  **severe cases** | Rapid diagnostic test per person | 0.55 | Global Fund Pooled Procurement Mechanism Reference Pricing (Q2 2023) (30),  Patouillard et al. 2017 (31) | Mean cost from “Reference price per test EXW, USD” column = $0.48. Includes 15% delivery mark-up based on Patouillard et al. 2017. | 27.99 per adult case  23.83 per child case  after removing 23% of treatment costs assumed to represent out-of-pocket payments (32) |
|  | Treatment with Artemether/Lumefantrine per dose | 0.45 | Global Fund Pooled Procurement Mechanism Reference Pricing: Antimalarial medicines (Q2 2023) (33),  WHO guidelines for malaria 2022 (34) | Mean cost for Artemether/Lumefantrine 20/120mg, “Reference price US$ per treatment”  24 doses for adults and 12 doses for children |  |
|  | Inpatient treatment per person | 25.0 | WHO CHOICE (35),  Patouillard et al. 2017 (31) | Median “Cost per inpatient bed-day, Results in 2010 USD, Model prediction” for all malaria-endemic countries in sub-Saharan Africa and across all types of hospitals = $6.08. Assuming an average 3-day stay based on Patouillard et al. 2017. Converted to 2023 US$. |  |
| **SMC** | Dose + delivery | 1.10 | Gilmartin et al. 2021 (36) | Based on the average economic cost of administering four monthly SMC cycles of sulfadoxine–pyrimethamine plus amodiaquine for children younger than 5 years across seven countries in the Sahel subregion using multiple delivery methods. Assuming four doses per cycle in highly seasonal settings and five doses in seasonal settings. Converted to 2023 US$. | 4.40 per full course in highly seasonal settings  5.50 per full course in seasonal settings |
| **RTS,S vaccine** | Dose | 10.20 | UNICEF Supply Division (37) | Cost for supply from GSK between 2023-2025. Full course includes 3 primary doses and 1 booster dose. | 61.20 per full course |
|  | Consumables per dose | 3.50 | Penny et al. 2016 (1),  Hutton et al. 2006 (38) | Includes infection and reconstitution syringes, the safety box, and accounts for freight and wastage. |  |
|  | Delivery cost per dose | 1.60 | Malaria Vaccine Implementation Programme (20) | Economic cost of delivery per dose, recurring only. Mean of the three countries in MVIP. |  |
| **Indoor residual spraying** | Cost per person protected | 7.80 | US President’s Malaria Initiative IRS country programs (39) | Mean from 16 African countries for long-lasting IRS products. | 7.80 per person protected |

## CHEERS-VOI reporting checklist

**Table S1.6. CHEERS-VOI checklist.**

| **Topic** | **Item no.** | **Guidance for reporting** | **Reported in section** |
| --- | --- | --- | --- |
| Title | 1 | Identify the study as an economic evaluation and *as a VOI analysis,* and specify the interventions being compared. | √ |
| Abstract | 2 | Provide a structured summary that highlights context, key methods, results, and alternative analyses. | √ |
| Introduction | | | |
| Background and objectives | 3 | Give the context for the study, the study question, and its practical relevance for decision making in policy or practice. | √ |
| Methods | | | |
| Health economic analysis plan | 4 | Indicate whether a health economic analysis plan was developed and where it is available. | Not developed |
| Study population | 5 | Describe characteristics of the study population (such as age range, demographics, socioeconomic, or clinical characteristics). *If population-level VOI measures are estimated, describe and justify how the population who benefits from the research was defined, how the incidence and/or prevalence were derived.* | Methods “Cost-effectiveness and VOI analysis” |
| Setting and location | 6 | Provide relevant contextual information that may influence findings. | Not applicable |
| Comparators | 7 | Describe the interventions or strategies being compared and why they were chosen. | Methods “Transmission settings and model scenarios” |
| Perspective | 8 | State the perspective(s) adopted by the study and why they were chosen. | Supplementary methods “Costing and cost-effectiveness thresholds” |
| Time horizon | 9 | State the time horizon for the study that is *assumed in the economic evaluation* and why it is appropriate. | Methods “Transmission settings and model scenarios” |
| Discount rate | 10 | Report the discount rate(s) *used in the economic evaluation and for the* *population-level* *VOI analysis* and the reason why they were chosen. | Supplementary methods “Costing and cost-effectiveness thresholds” |
| Selection of outcomes | 11 | Describe what outcomes were used as the measure(s) of benefit(s) and harm(s). | Methods “Cost-effectiveness and VOI analysis”, Supplementary methods “Transmission model”, “Calculation of DALYs” |
| Measurement of outcomes | 12 | Describe how outcomes used to capture benefit(s) and harm(s) were measured. |  |
| Valuation of outcomes | 13 | Describe the population and methods used to measure and value outcomes. |  |
| Measurement and valuation of resources and costs | 14 | Describe how costs were valued. | Supplementary methods “Costing and cost-effectiveness thresholds” |
| Currency, price date, and conversion | 15 | Report the dates of the estimated resource quantities and unit costs, plus the currency and year of conversion. | Supplementary methods “Costing and cost-effectiveness thresholds” |
| Rationale and description of model | 16 | If modelling is used, describe in detail and why used. *Describe the model structure and justify structural assumptions that have been made.* Report if the model is publicly available and where it can be accessed. | Supplementary methods “Transmission model” |
| VOI Estimation Methods | S1 | (a) Describe the method used to estimate EVPI and any checks used to determine its accuracy. (b) Describe the method used to estimate EVPPI and any checks used to determine its accuracy. (c) Describe the method(s) used to estimate EVSI and any checks used to determine accuracy. | Methods “Cost-effectiveness and VOI analysis” |
| Analytics and assumptions | 17 | Describe any methods for analyzing or statistically transforming data, any extrapolation methods, and approaches for validating any model used. *Make sure to do the following: (a) State and justify the* *cost-effectiveness* *threshold(s) chosen*. *(b) Describe and justify the statistical and methodological choices that were made to estimate parameters (eg, methods of data synthesis and calibration)*. | Methods “Sources of entomological parameter uncertainty”, “Cost-effectiveness and VOI analysis”, Supplementary methods “Costing and cost-effectiveness thresholds” |
| Evidence base | S2 | Discuss the potential risk of bias and heterogeneity in the evidence base and describe whether these were adjusted for in the model. | Methods “Sources of entomological parameter uncertainty”, Discussion |
| Characterizing heterogeneity | 18 | Describe any methods used for estimating how the results of the study vary for subgroups. | Not applicable |
| Characterizing distributional effects | 19 | Describe how impacts are distributed across different individuals or describe adjustments made to reflect priority populations. | Not applicable |
| Characterizing uncertainty | 20 | Describe methods to characterize any sources of uncertainty in the analysis. *Discuss the key structural uncertainties and how these have been addressed. For parameters subject to a probabilistic analysis, describe how the distributions reflecting uncertainty were derived, including any dependencies between parameters. For parameters that were not subject to a probabilistic analysis, explain why they are considered fixed and known with certainty*. | Methods “Sources of entomological parameter uncertainty”, “Cost-effectiveness and VOI analysis”, Supplementary methods “Transmission model” |
| Parameters of Interest in VOI analysis | S3 | (a) Specify the individual and/or combinations of parameters for which EVPPI has been computed. (b) Specify the individual and/or combinations of parameters for which EVSI has been computed. | Methods “Cost-effectiveness and VOI analysis” |
| Study Design(s) proposed in VOI analysis | S4 | Describe and justify the design of all proposed future research studies and indicate the model parameters that will be updated by these studies. | Not applicable |
| Data Generation for EVSI | S5 | Describe the assumptions used to generate the study data. | Not applicable |
| Costs of Research Studies for ENBS | S6 | Provide the costs of research studies proposed in VOI analysis (eg, fixed costs, variable costs, and opportunity costs of allocation), and describe how they were calculated. | Not applicable |
| Approach to engagement with patients and others affected by the study | 21 | Describe any approaches to engage patients or service recipients, the general public, communities, or stakeholders (such as clinicians or payers) in the design of the study. | Not applicable |
| Results | | | |
| Study parameters | 22 | Report all analytic inputs (such as values, ranges, and references), including uncertainty or distributional assumptions. | Methods “Sources of entomological parameter uncertainty”, Supplementary methods “Transmission model”, “Entomological efficacy parameter sets”, “Costing and cost-effectiveness thresholds” |
| Summary of main results | 23 | Report the mean values for the main categories of costs and outcomes of interest, and summarize them in the most appropriate overall measure. *Report the values for the VOI measures considered*. | Results |
| Effect of uncertainty | 24 | Describe how uncertainty about analytic judgments, inputs, or projections affect findings. Report the effect of choice of discount rate and time horizon, if applicable. | Results, Supplementary results “Sensitivity analysis on costs” |
| Effect of engagement with patients and others affected by the study | 25 | Report on any difference patient/service recipient, general public, community, or stakeholder involvement made to the approach or findings of the study. | Not applicable |
| Discussion | | | |
| Study findings, limitations, generalizability, and current knowledge | 26 | Report key findings, limitations, and ethical or equity considerations that are not captured and how these could affect patients, policy, or practice. | √ |
| Other relevant information | | | |
| Source of funding | 27 | Describe how the study was funded and any role of the funder in the identification, design, conduct, and reporting of the analysis. | √ |
| Conflicts of interest | 28 | Report authors conflicts of interest according to journal or International Committee of Medical Journal Editors requirements. | √ |

CHEERS-VOI indicates Consolidated Health Economic Evaluation Reporting Standards Value of Information; ENBS, expected net benefit of sampling; EVPI, expected value of perfect information; EVPPI, expected value of partial perfect information; EVSI, expected value of sample information; no., number.

# Supplementary results


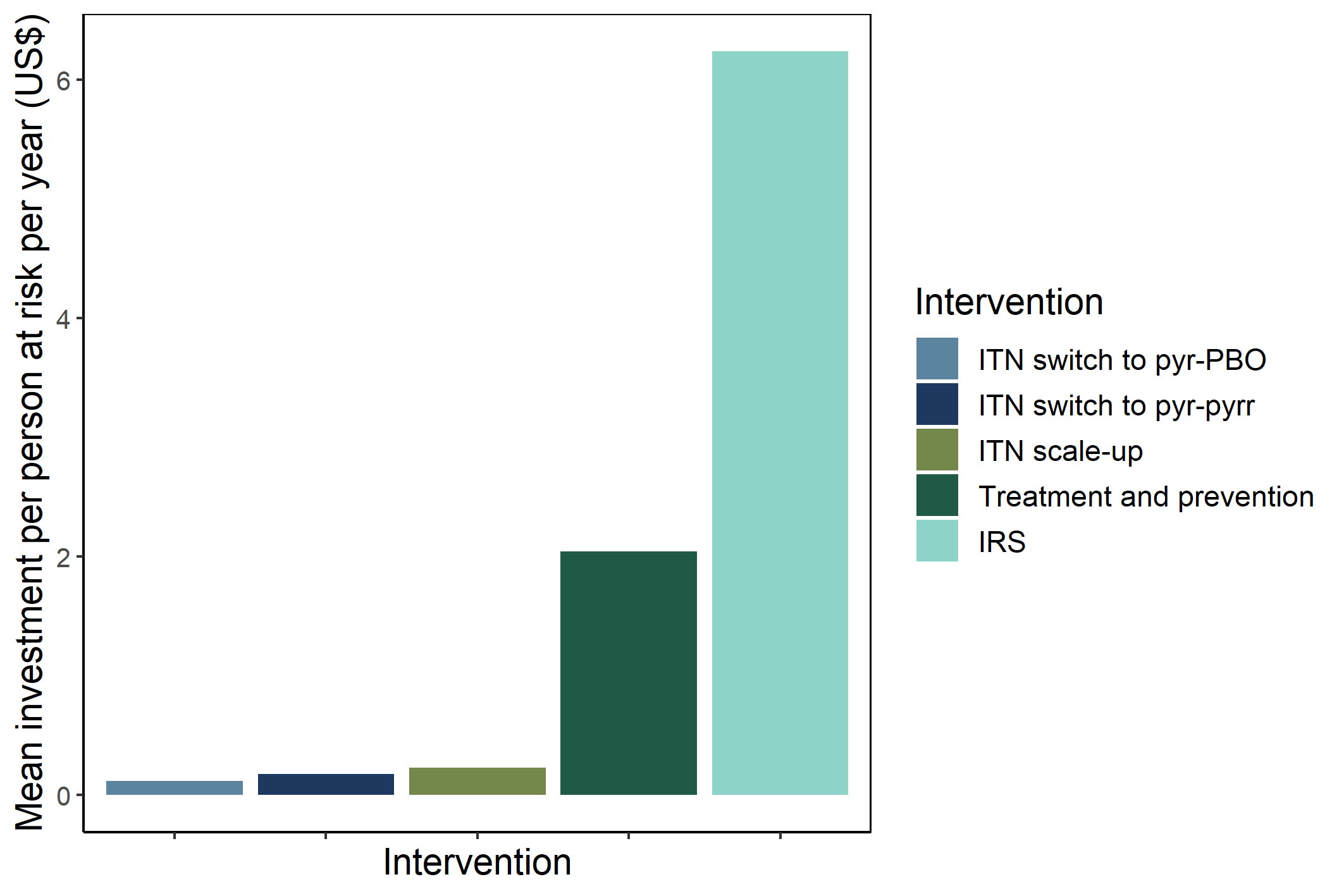


**Figure S2.1. Mean cost of introducing individual interventions per person at risk per year (excluding cost savings) over a 3-year period.** The investment associated with each intervention was averaged across all transmission settings and model simulations and also reflects the modelled coverage of the different interventions (e.g. between 20-60% ITN usage for “ITN switch to pyr-PBO” and 80% IRS). ITN = insecticide-treated net, pyr = pyrethroid, PBO = piperonyl butoxide, pyrr = pyrrole, IRS = indoor residual spraying.


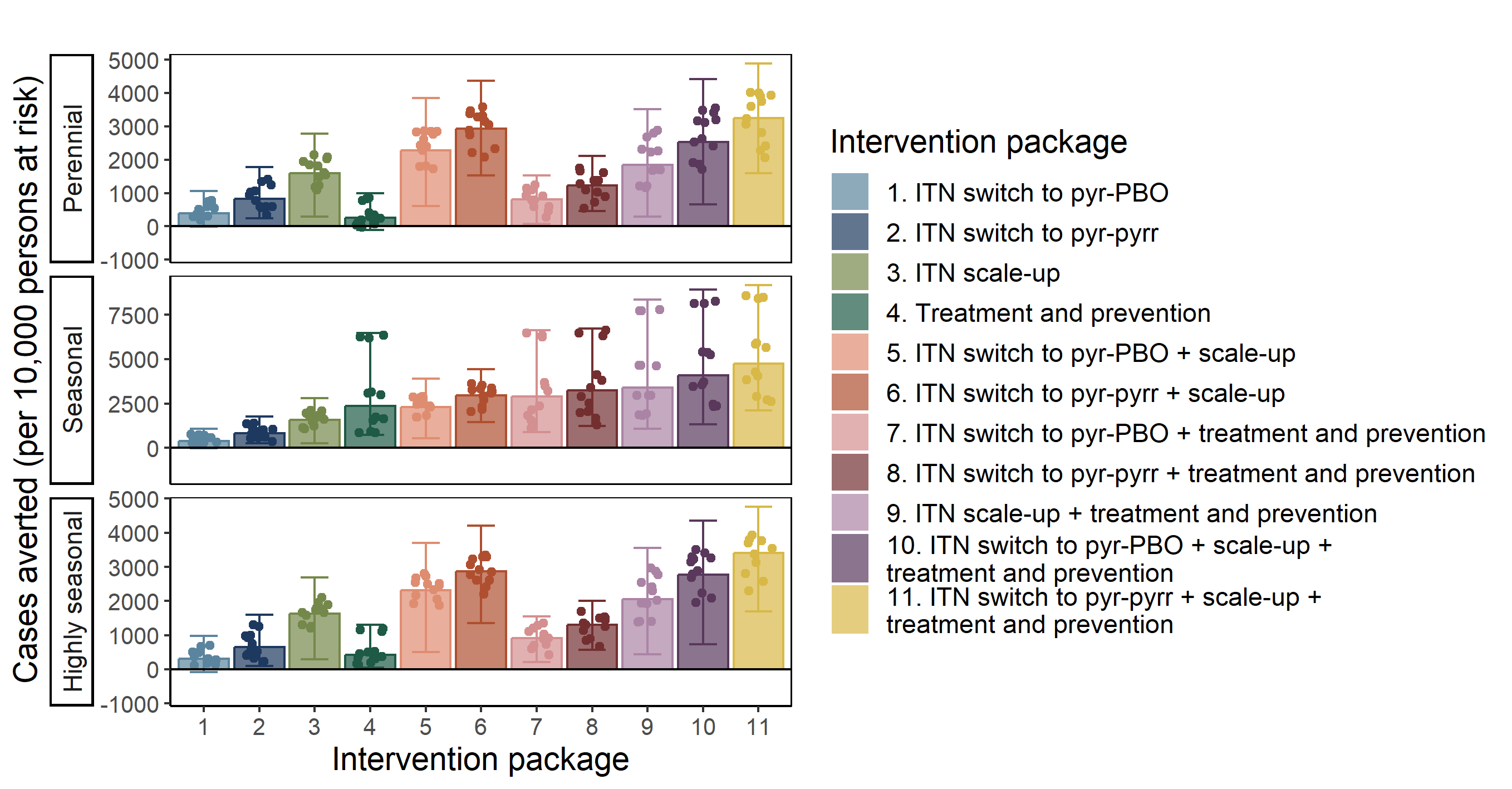


**Figure S2.2. Cases averted by the different intervention packages relative to the respective baseline setting stratified by seasonality.** Bars represent the median across settings (including *Pf*PR_2-10_ 5% to 40% and ITN use 20% to 60%), with error bars indicating the full range of values (across settings and parameter variation). Points represent the median value in each prevalence and baseline ITN use setting. The “prevention and treatment” package consists of a combination of treatment scale-up, SMC and/or RTS,S vaccination depending on the setting.


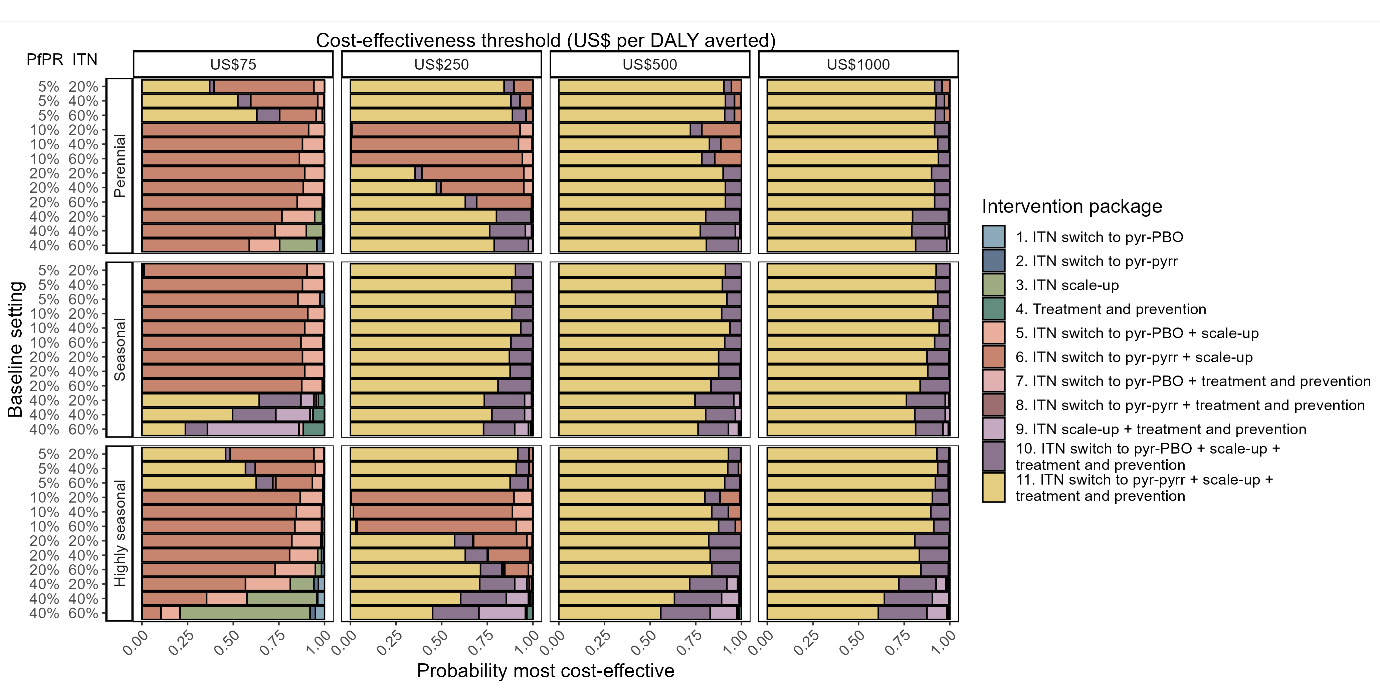


**Figure S2.3.** **Probability of each intervention package being the most cost-effective (having the highest mean net monetary benefit) in each setting if IRS is not considered (case A).** Results are shown for cost-effectiveness thresholds between US$75 and US$1000 per DALY averted. Settings are defined by their seasonality, parasite prevalence in 2-10 year olds (PfPR) and baseline insecticide-treated net usage (ITN).


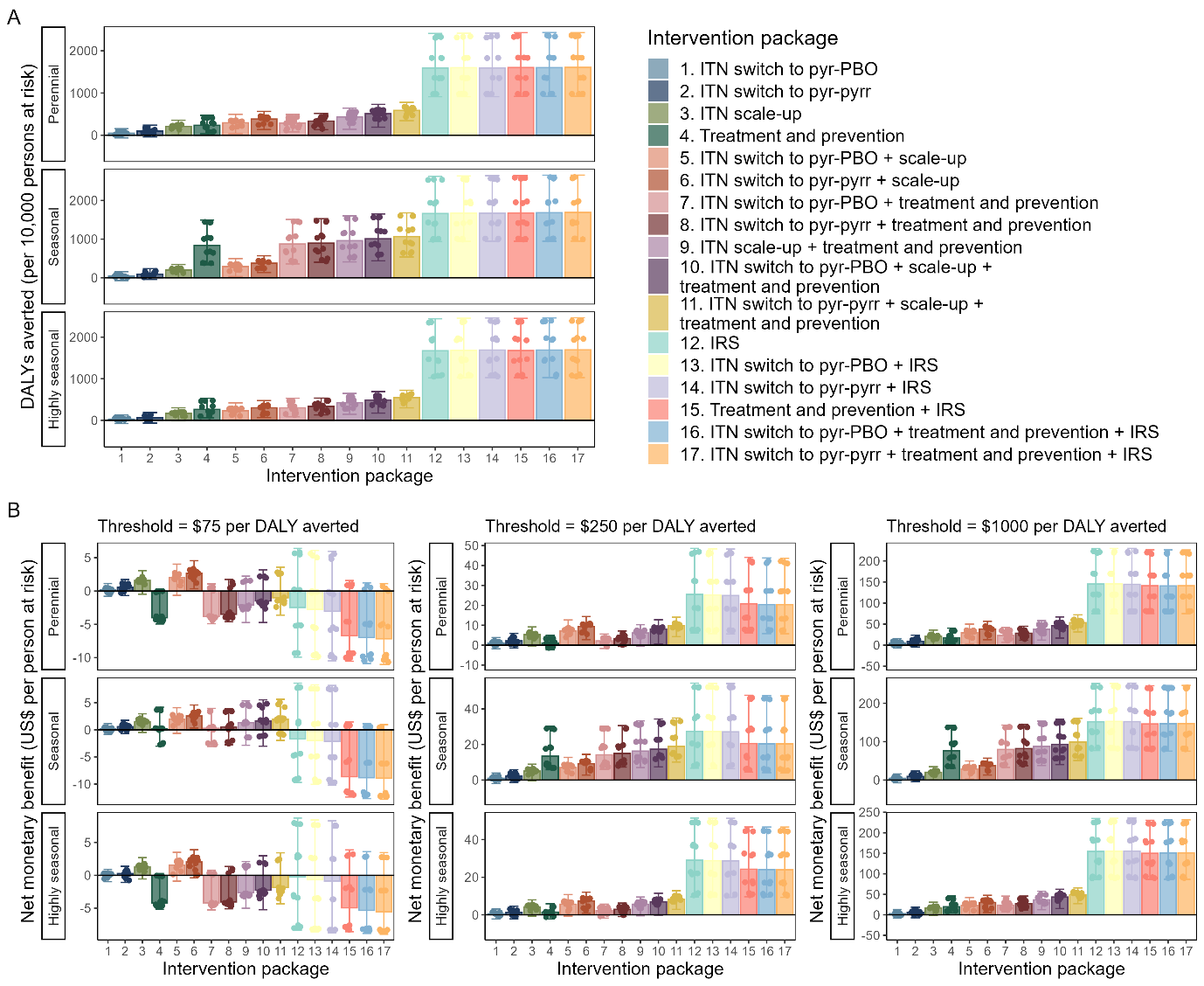


**Figure S2.4. Impact and cost-effectiveness of the different intervention packages including IRS (case B) relative to the corresponding baseline setting with existing interventions, stratified by seasonality.** Bars represent the median across settings (including *Pf*PR_2-10_ 5% to 40% and ITN use 20% to 60%), with error bars indicating the full range of values (across settings and parameter variation). Points represent the median value in each prevalence and baseline ITN use setting. The “prevention and treatment” package consists of a combination of treatment scale-up, SMC and/or RTS,S vaccination depending on the setting. A) DALYs averted by the different intervention packages. B) Net monetary benefit (NMB) of the different intervention packages at cost-effectiveness thresholds of US$75, US$250 and US$1000 per DALY averted. A positive NMB indicates that the intervention is cost-effective compared to existing interventions at baseline; the optimal intervention package is the one with the highest average NMB. Calculation of the NMB depends on assumptions about the value of an averted DALY, therefore NMBs are higher for a cost-effectiveness threshold of $1000 per DALY averted than the $250 threshold.


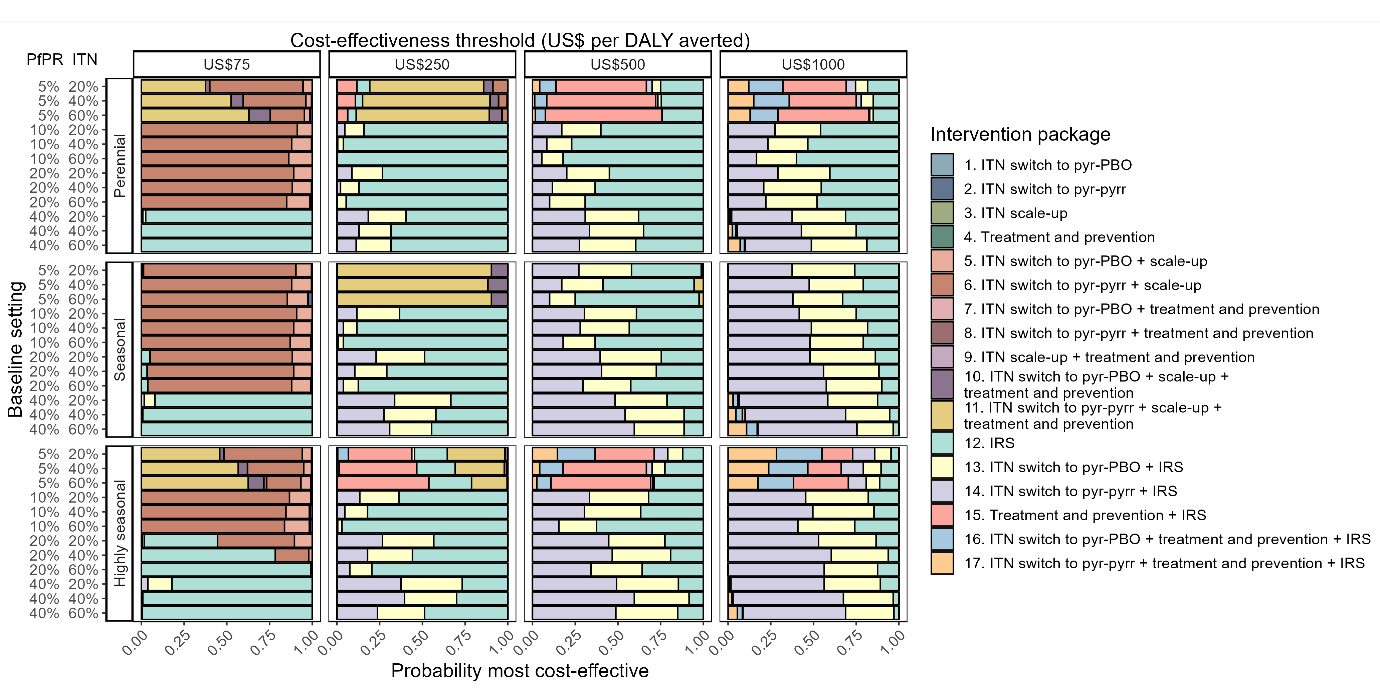


**Figure S2.5.** **Probability of each intervention package being the most cost-effective (having the highest mean net monetary benefit) in each setting if IRS is included as an option (case B).** Results are shown for cost-effectiveness thresholds between US$75 and US$1000 per DALY averted. Settings are defined by their seasonality, parasite prevalence in 2-10 year olds (PfPR) and baseline insecticide-treated net usage (ITN).

## Sensitivity analysis on costs

Varying costs of individual or groups of interventions between half and double the default values showed that the EVPI was most sensitive to cost assumptions at the lowest cost-effectiveness thresholds, with little effect at the highest threshold of US$3000 per DALY averted (**Figure S2.6**). For thresholds of US$250 and over, the EVPI was most sensitive to the relative costs of pyrethroid-PBO and pyrethroid-pyrrole ITNs. Decision uncertainty was almost completely removed in many settings if the cost of pyrethroid-pyrrole ITNs was halved or the cost of pyrethroid-PBO ITNs was doubled with all other costs held constant (i.e. pyrethroid-pyrrole ITNs becoming cheaper than pyrethroid and pyrethroid-PBO ITNs or pyrethroid-PBO ITNs becoming more expensive than pyrethroid-pyrrole ITNs), and vice versa. At a cost-effectiveness threshold of US$75 per DALY averted, EVPI estimates additionally depended on varying the cost of all classes of ITNs together, all interventions, treatment and all prevention interventions.

**Figure S2.7** confirms that among different combination of ITN costs between US$2 and US$7, the median EVPI was most strongly influenced by the combination of pyrethroid-pyrrole and pyrethroid-PBO ITNs. The median EVPI would be lower than estimated under default assumptions if the cost of pyrethroid-pyrrole ITNs was reduced while the cost of pyrethroid-PBO ITNs stayed the same or increased. Decision uncertainty would be higher if the cost of pyrethroid-PBO ITNs was further reduced compared to that of pyrethroid-pyrrole ITNs.


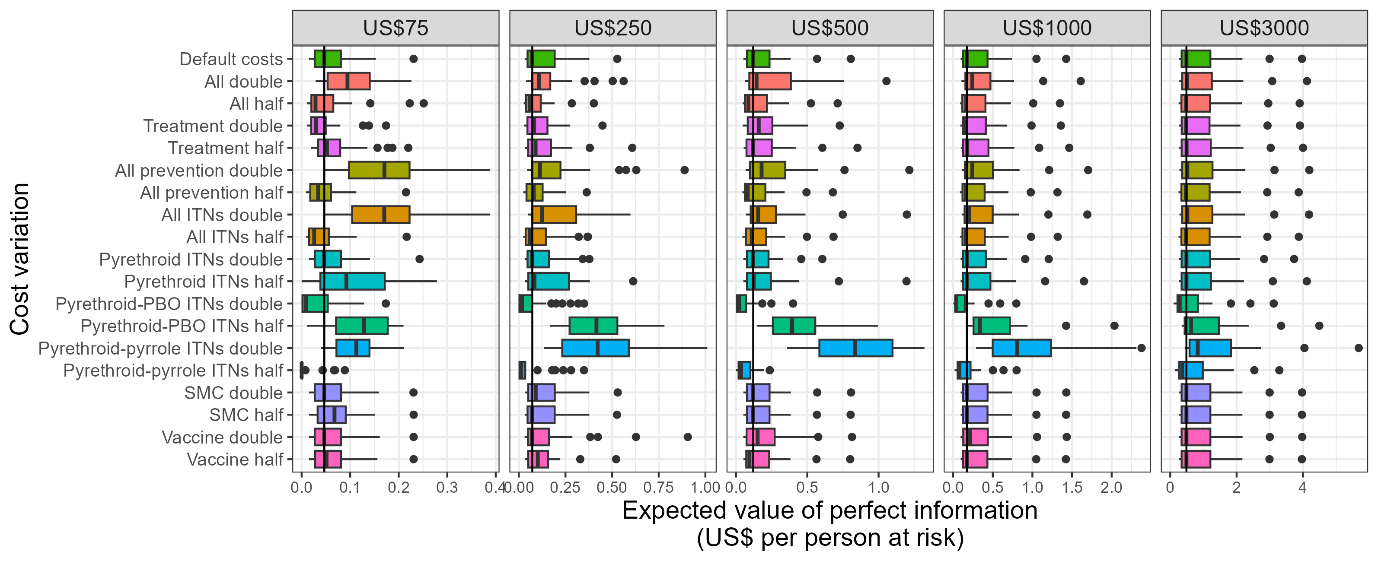
**Figure S2.6. Sensitivity analysis on intervention costs for cost-effectiveness thresholds between US$75 and US$3000 per DALY averted (case A without indoor residual spraying).** The top green bar shows the distribution of the expected value of perfect information across settings under the default cost assumptions in Table S1.5. In the other rows, the total cost of individual or groups of interventions were doubled or halved in the following order (with all other costs held constant): all interventions, treatment (of clinical and severe cases), all prevention interventions [all insecticide-treated nets (ITNs), seasonal malaria chemoprevention (SMC), vaccination)], all ITNs (pyrethroid, pyrethroid-PBO and pyrethroid-pyrrole), pyrethroid ITNs, pyrethroid-PBO ITNs, pyrethroid-pyrrole ITNs, SMC, and RTS,S vaccination.


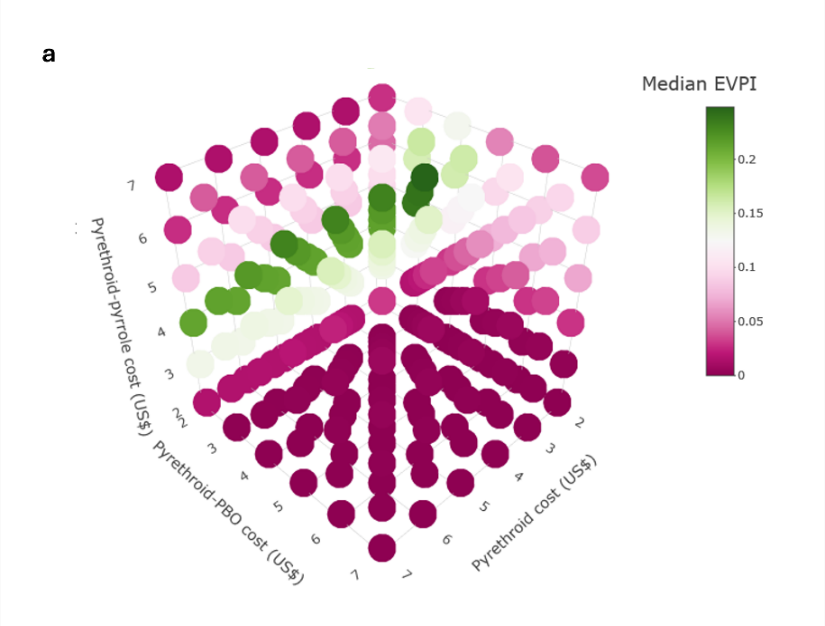

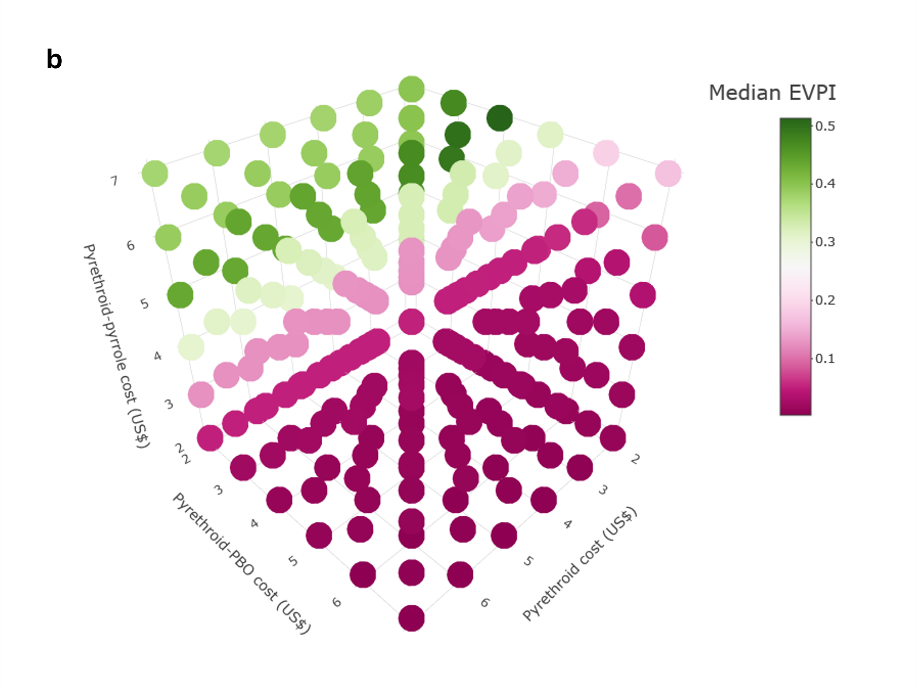

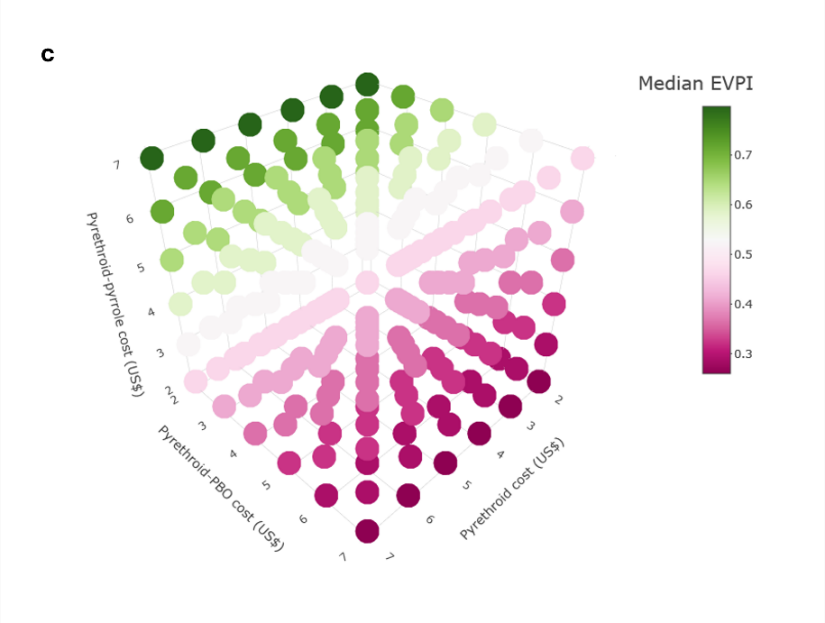


**Figure S2.7. Sensitivity analysis on insecticide-treated net costs for cost-effectiveness thresholds of US$75 (a), US$250 (b) and US$3000 (c) per DALY averted (case A without indoor residual spraying).** The colour scale shows how the median expected value of perfect information (EVPI) across settings varies for different combinations of unit costs of a pyrethroid, pyrethroid-PBO and pyrethroid-pyrrole net between US$2 and US$7. For the default cost assumption, the median EVPI was US$0.05 (a), US$0.07 (b) and US$0.49 (c) per person at risk.

# References

1. Penny MA, Verity R, Bever CA, Sauboin C, Galactionova K, Flasche S, et al. Public health impact and cost-effectiveness of the RTS, S/AS01 malaria vaccine: a systematic comparison of predictions from four mathematical models. The Lancet. 2016;387(10016):367-75.

2. Winskill P, Slater HC, Griffin JT, Ghani AC, Walker PG. The US President's Malaria Initiative, Plasmodium falciparum transmission and mortality: A modelling study. PLoS Medicine. 2017;14(11):e1002448.

3. Griffin JT, Bhatt S, Sinka ME, Gething PW, Lynch M, Patouillard E, et al. Potential for reduction of burden and local elimination of malaria by reducing Plasmodium falciparum malaria transmission: a mathematical modelling study. The Lancet Infectious Diseases. 2016;16(4):465-72.

4. Mousa A, Al-Taiar A, Anstey NM, Badaut C, Barber BE, Bassat Q, et al. The impact of delayed treatment of uncomplicated P. falciparum malaria on progression to severe malaria: A systematic review and a pooled multicentre individual-patient meta-analysis. PLoS Med. 2020;17(10):e1003359.

5. Griffin JT, Hollingsworth TD, Okell LC, Churcher TS, White M, Hinsley W, et al. Reducing Plasmodium falciparum malaria transmission in Africa: a model-based evaluation of intervention strategies. PLoS Medicine. 2010;7(8).

6. Okell LC, Cairns M, Griffin JT, Ferguson NM, Tarning J, Jagoe G, et al. Contrasting benefits of different artemisinin combination therapies as first-line malaria treatments using model-based cost-effectiveness analysis. Nature communications. 2014;5(1):5606.

7. Sherrard-Smith E, Griffin JT, Winskill P, Corbel V, Pennetier C, Djénontin A, et al. Systematic review of indoor residual spray efficacy and effectiveness against Plasmodium falciparum in Africa. Nature communications. 2018;9(1):4982.

8. White MT, Verity R, Griffin JT, Asante KP, Owusu-Agyei S, Greenwood B, et al. Immunogenicity of the RTS, S/AS01 malaria vaccine and implications for duration of vaccine efficacy: secondary analysis of data from a phase 3 randomised controlled trial. The Lancet infectious diseases. 2015;15(12):1450-8.

9. Thompson HA, Hogan AB, Walker PG, Winskill P, Zongo I, Sagara I, et al. Seasonal use case for the RTS, S/AS01 malaria vaccine: a mathematical modelling study. The Lancet Global Health. 2022;10(12):e1782-e92.

10. White MT, Griffin JT, Churcher TS, Ferguson NM, Basáñez M-G, Ghani AC. Modelling the impact of vector control interventions on Anopheles gambiae population dynamics. Parasites & vectors. 2011;4:1-14.

11. Winskill P, Walker PG, Cibulskis RE, Ghani AC. Prioritizing the scale-up of interventions for malaria control and elimination. Malaria Journal. 2019;18(1):1-11.

12. Aghajanyan A, Riley M, Tesso E, Won N, Dawadi S, Sanchez A, et al. PMI IRS Country Programs: 2022 Comparative Cost Analysis. Rockville, MD: PMI VectorLink Project, Abt Associates Inc.; 2023.

13. Zongo I, Milligan P, Compaore YD, Some AF, Greenwood B, Tarning J, et al. Randomized Noninferiority Trial of Dihydroartemisinin-Piperaquine Compared with Sulfadoxine-Pyrimethamine plus Amodiaquine for Seasonal Malaria Chemoprevention in Burkina Faso. Antimicrob Agents Chemother. 2015;59(8):4387-96.

14. Chandramohan D, Zongo I, Sagara I, Cairns M, Yerbanga R-S, Diarra M, et al. Seasonal malaria vaccination with or without seasonal malaria chemoprevention. New England Journal of Medicine. 2021;385(11):1005-17.

15. RTSS Clinical Trials Partnership. Efficacy and safety of RTS, S/AS01 malaria vaccine with or without a booster dose in infants and children in Africa: final results of a phase 3, individually randomised, controlled trial. The Lancet. 2015;386(9988):31-45.

16. Malaria Atlas Project. Pf infection prevalence (subnational) 2022 [Available from: <https://data.malariaatlas.org>.

17. Malaria Atlas Project. ITN use (subnational) 2022 [Available from: <https://data.malariaatlas.org>.

18. World Health Organization. WHO Guidelines for malaria, 16 October 2023. Geneva: World Health Organization; 2023.

19. Topazian HM, Schmit N, Gerard-Ursin I, Charles GD, Thompson H, Ghani AC, et al. Modelling the relative cost-effectiveness of the RTS, S/AS01 malaria vaccine compared to investment in vector control or chemoprophylaxis. Vaccine. 2023;41(20):3215-23.

20. Malaria Vaccine Implementation Programme (MVIP) Programme Advisory Group. Full Evidence Report on the RTS,S/AS01 Malaria Vaccine Geneva: World Health Organization; 2021 [Available from: <https://cdn.who.int/media/docs/default-source/immunization/mvip/full-evidence-report-on-the-rtss-as01-malaria-vaccine-for-sage-mpag-%28sept2021%29.pdf>.

21. Sherrard-Smith E, Winskill P, Hamlet A, Ngufor C, N'Guessan R, Guelbeogo MW, et al. Optimising the deployment of vector control tools against malaria: a data-informed modelling study. The Lancet Planetary Health. 2022;6(2):e100-e9.

22. Churcher TS, Stopard IJ, Hamlet A, Dee DP, Sanou A, Rowland M, et al. The epidemiological benefit of pyrethroid–pyrrole insecticide treated nets against malaria: an individual-based malaria transmission dynamics modelling study. Lancet Global Health. 2024;12(12):e1973-e83.

23. World Health Organization. Life expectancy at birth (years) [Data table] Geneva2020 [Available from: <https://www.who.int/data/gho/data/indicators/indicator-details/GHO/life-expectancy-at-birth-(years>).

24. Global Burden of Disease Collaborative Network. Global Burden of Disease Study 2017 (GBD 2017) Disability Weights. Seattle, United States of America: Institute for Health Metrics and Evaluation (IHME); 2018.

25. Wilkinson T, Sculpher MJ, Claxton K, Revill P, Briggs A, Cairns JA, et al. The International Decision Support Initiative Reference Case for Economic Evaluation: An Aid to Thought. Value Health. 2016;19(8):921-8.

26. Turner HC, Lauer JA, Tran BX, Teerawattananon Y, Jit M. Adjusting for Inflation and Currency Changes Within Health Economic Studies. Value Health. 2019;22(9):1026-32.

27. Schmit N, Topazian HM, Natama HM, Bellamy D, Traoré O, Somé MA, et al. The public health impact and cost-effectiveness of the R21/Matrix-M malaria vaccine: a mathematical modelling study. Lancet Infect Dis. 2024;24(5):465-75.

28. Ochalek J, Lomas J, Claxton K. Estimating health opportunity costs in low-income and middle-income countries: a novel approach and evidence from cross-country data. BMJ Glob Health. 2018;3(6):e000964.

29. The Global Fund. Pooled Procurement Mechanism Reference Pricing: Insecticide-Treated Nets, version: quarter 2, 2023 Geneva2023 [Available from: <https://www.theglobalfund.org/en/sourcing-management/health-products/long-lasting-insecticidal-nets/>.

30. The Global Fund. Pooled Procurement Mechanism Reference Pricing: RDTs, version: quarter 2, 2023 Geneva2022 [Available from: <https://www.theglobalfund.org/media/7564/psm_hivrdtreferencepricing_table_en.pdf>.

31. Patouillard E, Griffin J, Bhatt S, Ghani A, Cibulskis R. Global investment targets for malaria control and elimination between 2016 and 2030. BMJ global health. 2017;2(2):e000176.

32. DHS-USAID. STATcompiler. The DHS Program 2022 [Available from: <https://www.statcompiler.com/en/>.

33. The Global Fund. Pooled Procurement Mechanism Reference Pricing: Antimalarial medicines, version: quarter 2, 2023 Geneva2022 [Available from: <https://www.theglobalfund.org/media/5812/ppm_actreferencepricing_table_en.pdf>.

34. World Health Organization. WHO Guidelines for malaria, 31 March 2022. Geneva: World Health Organization; 2022. Contract No.: Geneva WHO/UCN/GMP/ 2022.01 Rev.1.

35. World Health Organization. Cost effectiveness and strategic planning (WHO-CHOICE) Geneva: World Health Organization; 2022 [Available from: <https://www.who.int/teams/health-systems-governance-and-financing/economic-analysis>.

36. Gilmartin C, Nonvignon J, Cairns M, Milligan P, Bocoum F, Winskill P, et al. Seasonal malaria chemoprevention in the Sahel subregion of Africa: a cost-effectiveness and cost-savings analysis. The Lancet Global Health. 2021;9(2):e199-e208.

37. UNICEF Supply Division. Malaria Vaccine: Questions and Answers on Vaccine Supply, Price, and Market Shaping October 2023 New York2023 [Available from: <https://www.unicef.org/supply/media/19456/file/Malaria%20-%20Vaccine%20-%20QA%20-%20October%202023%20-%20English%20.pdf>.

38. Hutton G, Tediosi F. The costs of introducing a malaria vaccine through the expanded program on immunization in Tanzania. Am J Trop Med Hyg. 2006;75(2 Suppl):119-30.

39. Longman B, Won N, Aghajanyan A, Sanchez A. PMI IRS Country Programs: 2021 Comparative Cost Analysis. Rockville, MD: PMI VectorLink Project, Abt Associates Inc.; 2022.
